# Mu-opioid receptor and receptor tyrosine kinase crosstalk: Implications in mechanisms of opioid tolerance, reduced analgesia to neuropathic pain, dependence, and reward

**DOI:** 10.3389/fnsys.2022.1059089

**Published:** 2022-12-01

**Authors:** Mackenzie C. Gamble, Benjamin R. Williams, Navsharan Singh, Luca Posa, Zachary Freyberg, Ryan W. Logan, Stephanie Puig

**Affiliations:** ^1^Department of Pharmacology and Experimental Therapeutics, Boston University School of Medicine, Boston, MA, United States; ^2^Molecular and Translational Medicine, Department of Medicine, Boston University School of Medicine, Boston, MA, United States; ^3^Department of Psychiatry, University of Pittsburgh School of Medicine, Pittsburgh, PA, United States; ^4^Department of Cell Biology, University of Pittsburgh School of Medicine, Pittsburgh, PA, United States; ^5^Center for Systems Neuroscience, Boston University, Boston, MA, United States

**Keywords:** mu-opioid receptor, opioid signaling, pain, tolerance, neuropathic pain, physical dependence, reward, receptor tyrosine kinase

## Abstract

Despite the prevalence of opioid misuse, opioids remain the frontline treatment regimen for severe pain. However, opioid safety is hampered by side-effects such as analgesic tolerance, reduced analgesia to neuropathic pain, physical dependence, or reward. These side effects promote development of opioid use disorders and ultimately cause overdose deaths due to opioid-induced respiratory depression. The intertwined nature of signaling via μ-opioid receptors (MOR), the primary target of prescription opioids, with signaling pathways responsible for opioid side-effects presents important challenges. Therefore, a critical objective is to uncouple cellular and molecular mechanisms that selectively modulate analgesia from those that mediate side-effects. One such mechanism could be the transactivation of receptor tyrosine kinases (RTKs) via MOR. Notably, MOR-mediated side-effects can be uncoupled from analgesia signaling via targeting RTK family receptors, highlighting physiological relevance of MOR-RTKs crosstalk. This review focuses on the current state of knowledge surrounding the basic pharmacology of RTKs and bidirectional regulation of MOR signaling, as well as how MOR-RTK signaling may modulate undesirable effects of chronic opioid use, including opioid analgesic tolerance, reduced analgesia to neuropathic pain, physical dependence, and reward. Further research is needed to better understand RTK-MOR transactivation signaling pathways, and to determine if RTKs are a plausible therapeutic target for mitigating opioid side effects.

## Introduction

The opioid epidemic has reached unprecedented proportions globally. In the United States alone, overdoses caused by opioids have claimed the lives of over hundreds of thousands of people, with rates of lethal overdoses expected to double in the next five years (Holland et al., 2021; Pickard and Lee, 2021). Prescription opioids are major contributors to the current opioid crisis, despite serving as the mainstay treatment for severe and chronic pain. Safe use of opioids is hampered by potentially severe side-effects including respiratory depression and the development of dependence and addiction (Benyamin et al., 2008; Pattinson, 2008; Henry et al., 2015; Hayhurst and Durieux, 2016; Algera et al., 2019). Emergence of these side-effects is promoted by escalating doses of opioids in chronic pain patients to mitigate the development of analgesic tolerance (Collett, 1998; Benyamin et al., 2008; Henry et al., 2015). High opioid doses are also necessary in neuropathic pain patients to overcome the minimal analgesic efficacy of current opioid-based therapies (Przewlocki and Przewlocka, 2001; Balayssac et al., 2009; Donica et al., 2014; Puig et al., 2020b). Such chronically high opioid doses promote physical dependence, causing deleterious physiological symptoms upon opioid withdrawal (Azolosa et al., 1994; Epstein et al., 2006; Burma et al., 2017), and ultimately prevents the discontinuation of opioid treatment. As a result, patients are forced to choose between effective pain treatments and the risk of physical dependence and/or addiction. With high doses, patients also risk developing respiratory depression (decreased respiration), the main cause of overdoses death (Pattinson, 2008; Algera et al., 2019). Opioid addiction has resulted in severe social and steep economic costs of hundreds of billions of dollars annually (The Council of Economic Advisers, 2017) and spurred a growing effort on finding new strategies to treat pain effectively and safely. One focus is toward finding a safe and “ideal” analgesic drug that would be free of addiction potentiating side-effects and have a low lethality. Unfortunately, to date, no safer alternative with equal analgesic efficacy to opioids has been found (Stuart et al., 2018). Many other proposed strategies involve reducing opioid dosage by locally targeting injured tissue (and limit central penetration), or reducing opioid prescriptions including establishing multimodal pain treatment regimens (as opposed to opioid monotherapy), opioid prescription monitoring, and restricted prescribing guidelines (Saloner et al., 2018; Mir et al., 2019; Franz et al., 2021). Yet this has not been enough. Therefore, it is imperative to continue efforts toward preserving long-term opioid analgesia, while mitigating side-effects. To this end, a better understanding of the molecular mechanisms underlying opioid signaling is needed.

Opioid receptors currently characterized include μ-opioid receptor (MOR), κ-opioid receptor (KOR), δ-opioid receptor (DOR), and opioid receptor like-1 (ORL1). These opioid receptors (ORs) belong to the class A (rhodopsin family) family of G protein-coupled receptors (GPCRs) which are coupled to inhibitory Gα_i/o_ G proteins. These GPCRs function to reduce neuronal excitability primarily by increasing potassium conductance and inhibiting voltage-gated calcium channels (Al-Hasani and Bruchas, 2011). Prescription opioids specifically modulate analgesia through MOR (Matthes et al., 1996; Loh et al., 1998), which is concentrated in structures essential for conductance of pain-related signaling including peripheral sensory neurons, spinal cord, brainstem and central brain nuclei (Mansour et al., 1994a,b, 1995a,1995b; Basbaum et al., 2009; Scherrer et al., 2009). Activation of MOR expressed on pain processing neurons via endogenous (e.g., endorphin) or exogenous (e.g., morphine or fentanyl) opioids directly inhibits these cells’ activity and controls analgesia (Al-Hasani and Bruchas, 2011).

Mechanisms of opioid analgesic tolerance and side-effects are still poorly understood (Adhikary and Williams, 2022). Traditionally, tolerance was thought to occur via the direct modulation of MOR signaling and trafficking (Williams et al., 2013). More recent evidence suggests that MOR-mediated side-effects can be uncoupled from analgesia, suggesting distinct signaling pathways for opioid-induced side effects versus analgesia (Puig and Gutstein, 2017; Paul et al., 2021). Separable pathways suggests that specific therapeutic strategies can be developed to selectively target side-effects without altering analgesia. This is further complicated by the fact that, apart from observational clinical studies, in contrast to animal experiments, practically no rigorously controlled clinical trials have unequivocally demonstrated pharmacodynamic tolerance to opioids in human patients (Collett, 1998; Henry et al., 2015), hampering the clinical translatability of earlier preclinical models.

Though the precise mechanisms for the above opioid-related signaling pathways remain to be determined, important clues have emerged which involve the receptor tyrosine kinase (RTK) family (Wang et al., 2012). More specifically, RTK signaling selectively regulates analgesic tolerance to MOR selective agonists (Puig et al., 2020a,b). Emerging evidence also suggests that RTKs could be involved in reduced opioid analgesia against neuropathic pain (Donica et al., 2014; Puig et al., 2020b), physical dependence (Rezamohammadi et al., 2020; Dorval et al., 2022), and reward (Koo et al., 2014; Fetterly et al., 2021). Together, these studies suggest that targeting opioid side-effects with RTK inhibitors could constitute a promising strategy to improve opioid safety. This review summarizes current knowledge about signaling interactions and crosstalk between MORs and RTKs. Furthermore, we discuss the implications of these mechanisms in opioid-mediated side-effects, with a focus on tolerance, reduced neuropathic pain analgesia, physical dependence, and reward. Finally, we discuss the potential clinical use of RTK inhibitors. Though RTK inhibitors are FDA-approved cancer chemotherapy drugs (Karaman et al., 2008; Gialeli et al., 2014; Roskoski, 2018), we present the possibility that these medications can be repurposed as a novel therapy for chronic pain and to improve opioid safety.

## Overview of mu-opioid receptor signaling

### Brief overview of mu opioid receptor signaling transduction pathways

**FIGURE 1:**
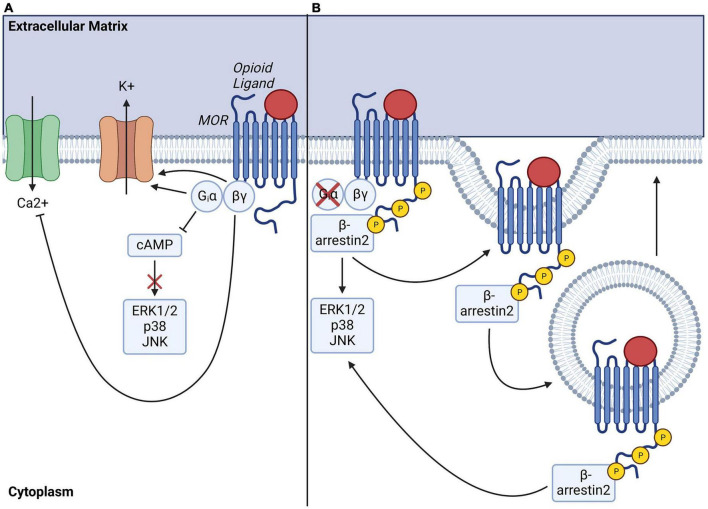
Mu-opioid receptor (MOR) signaling transduction pathways, internalization, and recycling. **(A)** Ligand-activation of MOR activates Gα_i/o_-induced inhibition of adenylate cyclase, resulting in decreased intracellular cAMP levels and depleted downstream signaling. Gα_i/o_ also serves to activate G protein gated inwardly rectifying potassium channels, leading to efflux of potassium ions while βγ heterodimers simultaneously perturb calcium influx by inhibiting voltage gated calcium channels, overall inhibiting intracellular signaling and cellular activity. **(B)** Ligand-activation of MOR also eventually leads to phosphorylation of MOR c-terminal tail by G protein receptor kinases (GRKs), which enables docking of β-arrestin2 and initiates MOR endocytosis for further receptor degradation or recycling. Note that recruitment of β-arrestin2 can also drive activation of downstream signaling effectors, including ERK, p38 or JNK pathways.

As a canonical GPCR, MOR recruits Gα_i/o_ G proteins upon stimulation. These inhibitory G proteins are composed of a monomeric α_i/o_ subunit and a dimeric G_βγ_ complex and are characterized by their sensitivity to pertussis toxin (Connor and Christie, 1999). At rest, the G proteins exist as an inactive G_α/βγ_ heterotrimeric complex that is GDP-bound. However, upon receptor activation by opioid ligands, changes in receptor conformation lead to the dissociation of G_α_ and G_βγ_ subunits via GDP/GTP exchange, which triggers intracellular signaling through downstream signaling effectors (Figure 1A). Canonical signaling pathways of Gα_i/o_ include inhibition of adenylyl cyclase (AC), the enzyme responsible for production of cyclic adenosine monophosphate (cAMP)—a critical second messenger of ORs. The resulting decrease in intracellular cAMP diminishes activity of protein kinase A (PKA) and PKA-dependent processes including activation of the C-AMP Response Element-binding protein (CREB) transcription factor. Gα_i/o_ signaling also positively regulates the activity of G protein-gated inwardly rectifying potassium (GIRK) channels, causing cellular hyperpolarization (Navarro et al., 1996). In parallel, G_βγ_ negatively regulates Ca^2+^ currents via inhibition of P/Q-type, N-type, or L-type Ca^2+^ channels, further contributing to overall inhibition of cellular activity (for review see: (Al-Hasani and Bruchas, 2011; Williams et al., 2013)). To illustrate, in pain circuitry, release of G_βγ_ subunits in presynaptic neurons results in inhibition of N-type Ca^2+^ channels for negatively modulating neurotransmitter release, while G_βγ_ subunits in postsynaptic neurons activate GIRKs, preventing neuronal depolarization (Chieng and Christie, 1994; Zamponi et al., 1997). Together, these mechanisms activated by MOR agonists result in analgesia via modulation of neuronal transmission in circuits conveying nociception. Following G protein signal transduction, G protein receptor kinases (GRKs) are recruited for phosphorylation of MOR on 11 potential phosphorylation sites present on the carboxyl terminal domain of the receptor, including serine (S), threonine (T), and tyrosine (Y) residues (Doll et al., 2011; Lau et al., 2011). Several GRKs (e.g., GRK2, GRK3, GRK5, GRK6) selectively phosphorylate different MOR phosphorylation sites, modulating signal transduction in a ligand and context-dependent manner (Lemel et al., 2020). Of note, other kinases, such as protein kinase C (PKC) or calcium/calmodulin-dependent protein kinase II (CaMKII), also phosphorylate MOR on selective phosphorylation sites in a ligand-dependent manner (Kelly et al., 2008). Additionally, MOR phosphorylation initiates receptor desensitization via receptor recruitment of β-arrestin2 (Figure 1B; Whistler and Von Zastrow, 1998; Martini and Whistler, 2007). This activates clathrin-mediated endocytosis of the MOR-β-arrestin2 complex, resulting in MOR internalization and recycling which terminates receptor signaling at the plasma membrane. The MOR-β-arrestin2 complex also recruits specific transduction signal proteins including kinases such as src, phosphoinositide 3-kinases (PI3K), or Mitogen-Activated Protein Kinases (MAPK), including extracellular signal-regulated kinases 1 and 2 (ERK 1 and 2), or c-Jun N- terminal Kinases (JNK) 1–3 (Pierce et al., 2001) (for full review of pathways see Williams et al., 2013; Jean-Charles et al., 2017). Finally, MOR signaling can be terminated by degradation via ubiquitination pathways (Chaturvedi et al., 2001; Petäjä-Repo et al., 2001).

### Proposed mechanisms of opioid-mediated side-effects

Mu-opioid receptor signaling is essential for opioids to induce analgesia and their side-effects, as global deletion of the gene encoding MOR (*Oprm1*) completely blocks opioid analgesia, reward, and physical dependence in rodents (Matthes et al., 1996; Loh et al., 1998). Indeed, most signaling pathways downstream of MOR are critical for the development and maintenance of opioid side-effects (Al-Hasani and Bruchas, 2011; Williams et al., 2013; Allouche et al., 2014; Zhou et al., 2021). Historically, mechanisms explaining side-effects of morphine were generalized to all MOR ligands, however, it has been difficult to find a single unifying mechanism that could explain side-effect profiles shared by all MOR agonists (Raehal and Bohn, 2011; Raehal et al., 2011; Whistler, 2012). This is likely since MOR ligands differ in their potencies, pharmacokinetics, and receptor internalization. Such drug-specific differences may also lead to varying recruitment of signaling effectors and pathways (Duttaroy and Yoburn, 1995; Keith et al., 1996, 1998; Trafton et al., 2000; Bohn et al., 2004; Kenakin, 2011; Posa et al., 2016; Schmid et al., 2017). Relatedly, different MOR ligands can stabilize the receptor in distinct conformations unique to each drug. As a result, different ligands can preferentially activate distinct signaling cascades that are biased toward either G protein versus β-arrestin2 pathways (Alvarez et al., 2002; Kenakin, 2011). Biased signaling downstream of MOR was proposed to drive the distinction between opioid side-effects and analgesia. In such a model, β-arrestin2 signaling preferentially mediates opioid-induced side effects while G protein signaling preferentially mediates the analgesic properties of these drugs (Bohn et al., 1999, 2000, 2002, 2003, 2004; Raehal and Bohn, 2011; Schmid et al., 2017). Consequently, much research has focused on identifying opioid ligands with higher intrinsic efficacy for stimulating G protein signaling downstream of MOR, while not triggering activation of the β-arrestin2 pathway (Soergel et al., 2014; Manglik et al., 2016). Although several G protein-biased compounds provide efficacious analgesia (Singla et al., 2019; Viscusi et al., 2019), adverse effects remain (Hill et al., 2018; Conibear and Kelly, 2019). Additionally, despite different signaling bias, all prescription opioids cause side-effects such as tolerance. G protein-biased MOR ligands thus cannot fully explain the mechanisms responsible for analgesia versus side-effects (Gillis et al., 2020).

Ultimately, a major thrust of opioid research is to uncouple the signaling mechanisms that selectively regulate analgesia from mechanisms that regulate undesired side-effects. New approaches proposing mechanisms that may not involve traditional canonical MOR signaling pathways may be key in addressing this issue. We propose that RTK signaling may be a common signaling pathway recruited downstream of MOR by all opioid agonists beyond their signaling bias. Here, we will present evidence suggesting that RTK signaling selectively modulates opioid side-effects but not analgesia. Therefore, we hypothesize that targeting RTKs offers a novel strategy to prevent and/or treat opioid side-effects without altering analgesia a critical objective for the field.

## Overview of receptor tyrosine kinase signaling

**FIGURE 2:**
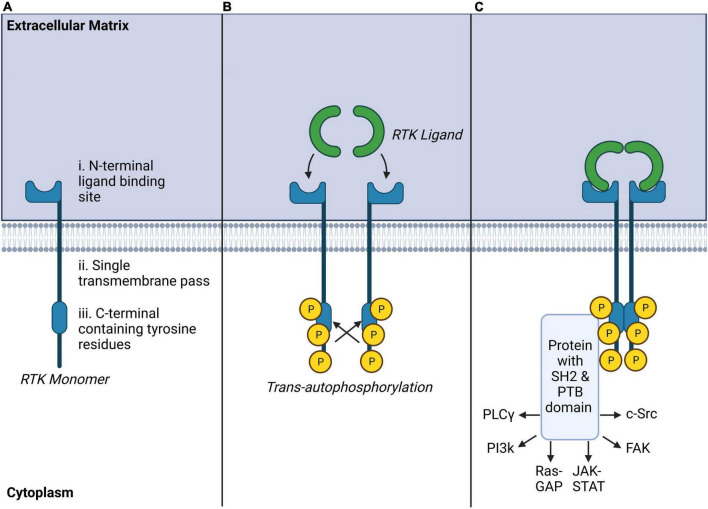
Receptor tyrosine kinase (RTK) structure, ligand binding and autophosphorylation, and common downstream signaling pathways. **(A)** RTK monomers are single transmembrane crossing peptides with extracellular ligand binding sites and tyrosine-rich intracellular effector regions. **(B)** RTK ligands bind as homo or heterodimers to RTKs inducing *trans*-autophosphorylation of opposing intracellular tyrosine residues. **(C)** Ligand-bound RTKs typically recruit protein complexes with SH2 and PTB domains which may activate a number of secondary intracellular messengers known to modulate other transmembrane receptors, intracellular signaling, or transcriptional regulation.

Receptor tyrosine kinases are a subclass of tyrosine kinases expressed at the cell surface which respond with high affinity to selective soluble polypeptide growth factors, cytokines, and hormones. RTKs constitute 20 sub-families (Robinson et al., 2000), including the ErbB family comprising the epidermal growth factor receptor (EGFR), platelet-derived growth factor receptor family (PDGFR), vascular-endothelial growth factor receptor family (VEGFR), tropomyosin receptor family (Trk), fibroblast growth factor receptor family (FGFR), ephrin receptor family (EphR), and insulin receptor family (IR) (Table 1). Structurally, RTKs are composed of single transmembrane glycoproteins, with the N-terminal extracellular domain containing the ligand-binding sequence, and the C-terminal intracellular domain containing multiple tyrosine residues which form the protein kinase catalytic core of these receptors (Du and Lovly, 2018; Figure 2). Ligand activation of RTKs elicits non-covalent oligomerization of monomeric RTKs and promotes formation of homo- or heterodimers. This process leads to *trans*-autophosphorylation (Honegger et al., 1989; Favelyukis et al., 2001) of key tyrosine residues on the interacting receptors. This activates downstream signaling via recruitment of selective docking proteins possessing Src homology-2 (SH2) and phosphotyrosine-binding (PTB) domains (Pawson, 2004); SH2 and PTB-domain-containing proteins include insulin receptor substrate-1 (IRS1), Grb2-associated binder (Gab1), and FGFR substrate 2 (FRS2α/FRS2β). These downstream proteins, lacking intrinsic kinase activity, serve as scaffolds to organize signaling complexes and trigger intracellular signaling cascades. Most docking proteins like Gab1 can be recruited by multiple RTKs. However, some are specific to a subset of receptors. For example, FRS2α and FRS2β are only involved in FGFR-, and Trk-mediated signaling (Schlessinger, 2000). This confers activation of specific signaling pathways by different subsets of RTKs and possibly enables signaling specificity. Pathways activated following docking protein recruitment include phospholipase Cγ (PLCγ), phosphoinositide 3-kinases (PI3K), mitogen-activated protein kinase/p38 (MAPK/p38), Ras-GTPase-activating protein (Ras-GAP), Janus kinase/signal transducer and activator of transcription (JAK/STAT), proto-oncogene c-Src, or focal adhesion kinase (FAK) signaling cascades (for a review see: (Lemmon and Schlessinger, 2010; Du and Lovly, 2018).

**TABLE 1:** Receptor tyrosine kinases identified to modulate opioid-mediated behaviors.

Receptor tyrosine kinase (RTK)	RTK-MOR crosstalk	Analgesic tolerance	Resistance of neuropathic pain to opioid analgesia	Opioid dependence	Opioid reward
Epidermal growth factor receptor (EGFR)	Belcheva et al., 2001; Belcheva et al., 2003; Belcheva et al., 2005; Miyatake et al., 2009; Zhao et al., 2013; Phamduong et al., 2014; Yang et al., 2021	Puig et al., 2020b	Martin et al., 2017; Puig et al., 2020b		
Fibroblast growth factor receptor (FGFR)		Fujita-Hamabe et al., 2011		Blackwood et al., 2019	
Platelet-derived growth factor receptor (PGFR)	Wang et al., 2012; Weber et al., 2013; Li et al., 2020	Wang et al., 2012; Puig and Gutstein, 2017; Puig et al., 2020a	Narita et al., 2005; Donica et al., 2014		
Insulin Receptor (IR)	Mclaughlin and Chavkin, 2001; Li et al., 2003				Li et al., 2003; Xu et al., 2012
Ephrin B	Liu et al., 2011	Liu et al., 2011	Han et al., 2008	Xia et al., 2014	
Tyrosine receptor kinase B (TrkB)				Peregud et al., 2016; Rezamohammadi et al., 2020	Freeman et al., 2003; Koo et al., 2012; Koo et al., 2014; Jorjani et al., 2021
Fms-like tyrosine kinase (FLT3)			Rivat et al., 2018		
Vascular endothelial growth factor receptor (VEGFR)		Lopez-Bellido et al., 2019			

Historically, RTK signaling pathways were found to be involved in cell proliferation, differentiation, migration, or metabolic changes (Lemmon and Schlessinger, 2010), and were also associated with cancer development (Du and Lovly, 2018). Most, if not all, RTK signaling effectors are also activated by opioid receptors. Numerous protein kinases including, ERK, JNK, p38, PKC, AKT, and CaMKII are utilized by both MOR and RTKs (Lemmon and Schlessinger, 2010; Williams et al., 2013). The ability of multiple receptors to concurrently activate signaling effectors raises the possibility of complex crosstalk between these receptors or even receptor cross-activation by the same molecule. Importantly, nearly all downstream pathways utilized by RTK receptors including MAP kinase cascades (Mckay and Morrison, 2007), PI3K (Haglund et al., 2007), PKC (Heckman and Wade, 2018), Akt (Choudhary et al., 2009), or ubiquitination (Haglund et al., 2003) play roles in opioid signaling in analgesia, tolerance, and dependence. It remains unclear, however, how these pathways pertain to opioid behaviors and side-effects (Mouledous et al., 2007; Chen et al., 2008a; Macey et al., 2009; Wang et al., 2009; Gregus et al., 2010). These discrepancies could be related to cellular context and, most importantly, they may involve modulation of signaling via differential engagement of RTK signaling in response to specific opioids.

## Mu-opioid receptors-receptor tyrosine kinases crosstalk

### General mechanisms of G protein-coupled receptors-receptor tyrosine kinases transactivation

**FIGURE 3:**
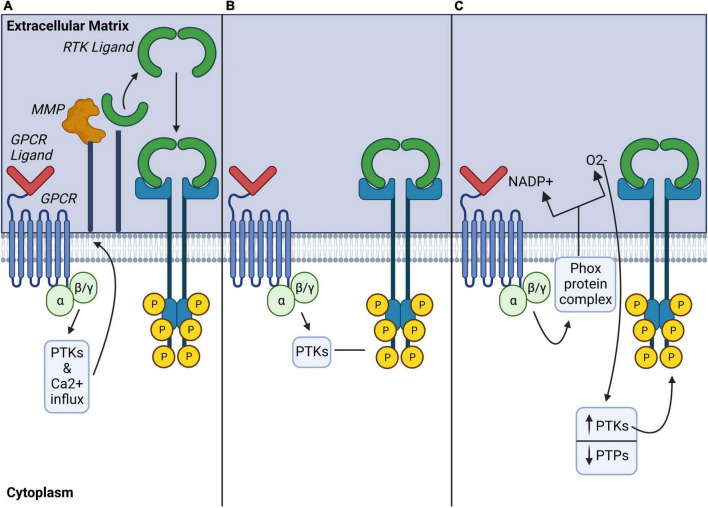
Ligand-dependent, ligand-independent, and atypical mechanisms of GPCR modulation of RTKs. **(A)** Ligand-dependent transactivation: Activated GPCRs induce a variety of downstream signaling pathways including activation of phospho-tyrosine kinases (PTKs), or increase of the influx of Ca^2+^, which activates matrix metalloproteinases (MMP) to cleave cell membrane-bound RTK ligands. **(B)** Ligand-independent transactivation: Activated GPCRs may also recruit intracellular PTKs to directly phosphorylate tyrosine residues on the intracellular domain of RTKs and induce their activation in a ligand-independent manner. **(C)** Atypical transactivation: Phox protein complexes activated by GPCRs generate reactive oxygen species which modulate phospho-tyrosine kinases (PTK) and phosphotyrosine phosphatases (PTP) activity to promote phosphorylation of intracellular RTK tyrosine residues. GPCR, G protein coupled receptor; MMP, matrix metalloproteinase; PTK, phosphotyrosine-kinase; PTP, phosphotyrosine phosphatases.

Crosstalk between GPCRs and RTKs can amplify signaling pathways downstream of one or both receptors in a process known as GPCR-RTK transactivation (Daub et al., 1996). This mechanism enables the integration of signal transduction between GPCRs and RTK signaling networks at signaling hubs shared between the respective receptor signaling pathways (Ragunathrao et al., 2019). Two major pathways of GPCR-RTK transactivation have been identified which involve either extracellular RTK ligand release (ligand-dependent) or intracellular recruitment of signaling effectors such as phosphotyrosine kinases (ligand-independent) (for review see Wetzker and Bohmer, 2003; Figure 3.

Ligand-dependent transactivation requires GPCR activation of matrix regulatory proteins such as membrane-bound matrix metalloproteinases (MMPs) or A Disintegrin and Metalloproteases (ADAMs) which contribute to the shedding of ligands. Several different MMPs or ADAMs are involved in the proteolytic ectodomain shedding of membrane bound RTK ligands from the extracellular matrix (ECM), which in turn, may transactivate several different RTKs (Cattaneo et al., 2014). This has been mostly described for EGFR as MMPs can cleave the heparin binding EGFR (Hb-EGF) to activate EGFR (Kilpatrick and Hill, 2021). Though the precise mechanisms of GPCR-mediated activation of MMPs or ADAMS are not fully understood, studies have implicated kinases such as c-src and PKC or calcium influx as activators of these proteases Figure 3A, for review, see Cattaneo et al. (2014). Notably, GPCR effectors like G_βγ_ (Overland and Insel, 2015) and β-arrestin2 (Noma et al., 2007; Oligny-Longpré et al., 2012).

Ligand-independent transactivation pathways involve complex intracellular signaling cascades which recruit kinases like Src or PI3K (Di Liberto et al., 2019) to phosphorylate selective tyrosine residues on RTKs (Figure 3B). This mode of GPCR-RTK transactivation can also require association of the two receptors via protein complex formation (for review see Wetzker and Bohmer, 2003). Of importance, GPCR-RTK heterodimerization may completely change GPCR signal transduction mechanisms and even promote a switch in the associated G protein. This is of particular interest because MOR signals via pertussis-toxin-insensitive stimulatory Gα_s_ proteins following chronic morphine exposure or neuropathic pain (Chakrabarti et al., 2005, 2010; Chakrabarti and Gintzler, 2007; Tsai et al., 2009). Therefore, involvement of RTKs in G protein switching downstream of MOR is a possibility that remains to be investigated.

Other ligand-independent transactivation involves atypical mechanisms of GPCR-RTK crosstalk via reactive oxygen species (ROS), such as nitric oxide (NO) (Figure 3C). ROS production by GPCRs could block protein-tyrosine phosphatases, activate phosphotyrosine kinases and modulate phosphorylation of RTK tyrosine residues (Cattaneo et al., 2014). Such a mechanism may be particularly relevant to opioid actions since ROS modulate MOR-mediated behaviors in rodents (Doyle et al., 2013). This therefore raises the possibility that RTKs could be involved in these ROS-mediated signaling pathways.

### Mu-opioid receptors-receptor tyrosine kinases transactivation *in vitro*

**FIGURE 4:**
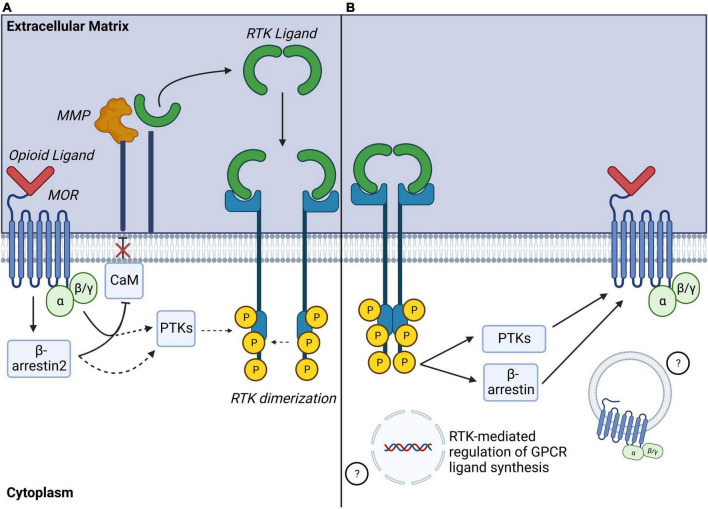
Identified mechanisms of MOR-RTK crosstalk. **(A)** Identified mechanisms of MOR-RTK transactivation: Activated MOR can induce MMP activation via mechanisms including disinhibition Calmodulin (CaM), leading to ligand shedding and ligand-dependent RTK activation. Other ligand-independent mechanisms may involve recruitment of intracellular phosphotyrosine kinases (PTKs) to phosphorylate RTK tyrosine residues. G protein and β-arrestin2 signaling may also be involved in MOR-RTK transactivation. **(B)** Identified mechanisms of RTK modulation of MOR signaling: Phosphorylated RTK may modulate activation of GPCR via a recruitment of PTKs, or β-arrestins activity. RTK activation may also lead to altered GPCR ligand gene expression or MOR internalization. MOR, mu-opioid receptor; MMP, matrix metalloproteinase; RTK, receptor tyrosine kinase; GPCR, G protein coupled receptor.

Most *in vitro* studies investigating RTK transactivation by MORs have focused on EGFR or PDGFRβ. In immortalized cell lines transfected with MOR, acute treatment with selective MOR agonists such as [D-Ala(2),MePhe(4),Gly-ol(5)]enkephalin (DAMGO) or morphine resulted in transactivation of EGFR (Belcheva et al., 2001, 2003, 2005; Phamduong et al., 2014) or PDGFRβ (Weber et al., 2013) as shown by phosphorylation of these RTKs. Interestingly, transactivation of RTKs by MOR activates downstream effector signaling at levels comparable to activation to direct activation of the RTKs themselves (Belcheva et al., 2001; Weber et al., 2013; Phamduong et al., 2014), and MOR-RTK transactivation can be abolished by pre-treatment with selective RTK inhibitors (Belcheva et al., 2001; Chen et al., 2008b; Weber et al., 2013). In cultured cells, mechanisms of MOR-EGFR and MOR-PDGFRβ transactivation were shown to require release of EGF (Belcheva et al., 2001, 2003, 2005; Phamduong et al., 2014) or of PDGF-B (Wang et al., 2012; Weber et al., 2013), respectively. Consistent with mechanisms of ligand-dependent GPCR-RTK transactivation, MOR-EGFR and MOR-PDGFRβ transactivation also require MMP activity (Belcheva et al., 2001). MMP activation by MOR may involve calmodulin (CaM), a Ca^2+^ sensor and binding protein. In resting conditions, CaM prevents MMP activity at the plasma membrane (PM) in HEK293 cells (Belcheva et al., 2001). Acute treatment with MOR agonist DAMGO promotes CaM translocation from the plasma membrane (PM) to MOR intracellular domains, lifting CaM inhibition on MMP via mechanisms involving activation of phospholipase C (PLC) and PKCε signaling (Belcheva et al., 2001, 2005; Miyatake et al., 2009; Figure 4A).

Other signaling effectors of MOR are involved in MOR-RTK transactivation. MOR-EGFR transactivation requires both Gα_i/o_ and β-arrestin2. Indeed, opioid-mediated EGFR phosphorylation can be attenuated via pertussis toxin or by siRNA-mediated β-arrestin2 silencing in cultured rat astrocytes (Miyatake et al., 2009). In addition to canonical MOR transduction pathways, other common signaling effectors between GPCRs and RTKs can take part in MOR-RTK transactivation. PI3K inhibitors abolish EGFR activation by DAMGO-activated MORs in cultured rat astrocytes, suggesting involvement of this kinase in MOR-RTK transactivation (Belcheva et al., 2005). Similarly, JNK inhibitors block MOR-PDGFRβ transactivation in rat spinal neurons (Li et al., 2020). Together, these studies indicate that MOR-RTK transactivation likely involves a complex network of converging signaling pathways (Figure 4A). It is important to note that most studies of mechanisms of MOR-RTK transactivation have employed acute MOR agonist treatments. However, longer MOR agonism may have different effects on RTK activity (Belcheva et al., 2003; Miyatake et al., 2009). Over hours, longer term treatment with MOR agonists DAMGO, enkephalin, or morphine induces EGFR phosphorylation as well as both downregulation and decreased ERK phosphorylation. These mechanisms are β-arrestin2- and Gα_i/o_-dependent and not observed with acute opioid treatments on the order of minutes, suggesting that acute versus longer-term events cause temporally distinct effects on signaling (Belcheva et al., 2003; Miyatake et al., 2009). Because most opioid-mediated side-effects occur after long-term opioid treatment, further studies to understand the specific alterations of MOR-RTK transactivation mechanisms by long-term opioid MOR stimulation are still needed.

Intriguingly, mechanisms of MOR-EGFR transactivation identified *in vitro* in immortalized cell lines do not differ between opioids with different ability to internalize MOR (Belcheva et al., 2001). Belcheva and colleagues (Belcheva et al., 2001) found that EGFR was phosphorylated by MOR whether it was activated by morphine (low internalizing (Sternini et al., 1996), DAMGO (highly internalizing synthetic opioid peptide (Keith et al., 1998) or endomorphin (highly internalizing endogenous opioid peptide (Mcconalogue et al., 1999). In addition, mechanisms of MOR-EGFR transactivation by these agonists all required similar mechanisms of CaM recruitment and PKC signaling, although they had been characterized as opioids with different signaling bias toward G protein and β-arrestin recruitment (Schmid et al., 2017). Together this implies that RTK transactivation mechanisms may be independent from MOR-ligands bias.

### Receptor tyrosine kinases transactivation of mu-opioid receptors

In addition to modulation of RTK signaling by GPCRs, RTKs can also modulate GPCR-mediated signaling, suggesting that the relationship between GPCRs and RTKs is reciprocal (Delcourt et al., 2007; Figure 4B). General mechanisms of GPCR transactivation by RTKs or “GPCR highjacking” (Delcourt et al., 2007) can involve recruitment of GPCR signaling effectors like GRKs (García-Sáinz et al., 2010; Sun et al., 2018), β-arrestins (Dalle et al., 2001; Povsic et al., 2003; Hupfeld and Olefsky, 2007) or activation of RTK downstream kinases including PI3K (Molina-Munþoz et al., 2006), Akt, or c-Src (Baltensperger et al., 1996; Doronin et al., 2002; Gavi et al., 2007). These mechanisms either require physical interactions between GPCRs and RTKs or transcriptional regulation of GPCR ligand synthesis (Delcourt et al., 2007). Relevant to this review, accumulating studies show that RTK signaling influences MOR signal transduction by modulation of phosphorylation. In cultured *Xenopus laevi* oocytes co-transfected with MOR and the insulin receptor (IR), pretreatment with insulin potentiated DAMGO-activated GIRK inward currents via MAPK signaling and possible dephosphorylation of MOR tyrosine residues, Y-106, or Y-166. Thus, indirectly demonstrating that IR signaling modulates MOR-signaling efficacy (Mclaughlin and Chavkin, 2001). In contrast, concomitant activation of MOR by DAMGO and activation of EGFR by EGF in HEK293 cells promotes MOR phosphorylation on Y-166 in a src-dependent manner, resulting in negative regulation of MOR-G protein coupling (Clayton et al., 2010). This suggests that regulation of MOR phosphorylation by opioids may be modulated by RTK-dependent activity. In separate studies, EGFR activation by EGF caused recruitment and translocation of G-coupled protein receptor kinase 2 (GRK-2) to the plasma membrane where it phosphorylated MOR on Serine-residues 363 and 375 (S-363, S-375), and Threonine-residue-370 (T-370), and enabled DAMGO-mediated MOR internalization (Chen et al., 2008b). Together, these studies highlight that RTKs can modulate MOR phosphorylation, signaling and internalization.

## Involvement of receptor tyrosine kinase signaling opioid-mediated behaviors

While much of the literature has focused on MOR-RTK transactivation *in vitro*, this phenomenon is also relevant physiologically *in vivo*, particularly in the development and maintenance of deleterious opioid side-effects caused by MOR agonists. Reviewed here is evidence that several RTKs (Table 1) play major roles in mediating opioid side-effects such as analgesic tolerance, resistance of neuropathic pain to opioid analgesia, physical dependence or reward.

### Receptor tyrosine kinase signaling and opioid analgesic tolerance

In the clinic, opioid analgesic tolerance is defined by a gradual loss of analgesic efficacy to a fixed dose of an opioid. As a result, escalation of opioid doses occurs over time to maintain analgesic benefit (Henry et al., 2015; Hayhurst and Durieux, 2016). MOR signaling is essential in the mechanisms of tolerance (Williams et al., 2013; Adhikary and Williams, 2022) and MOR is expressed in structures strongly implicated in tolerance and pain mechanisms including dorsal root ganglia (DRG) neurons and neurons of the spinal cord substantia gelatinosa (Mansour et al., 1988, 1995a; Scherrer et al., 2009; Corder et al., 2017; Puig and Gutstein, 2017). RTKs are similarly expressed alongside MOR in the spinal cord and DRG, including PDGFRβ (Sasahara et al., 1991; Eccleston et al., 1993), EGFR (Werner et al., 1988; Huerta et al., 1996), VEGFR2 (Spliet et al., 2004; Herrera et al., 2009), or Ephrin type-B receptor 1 (EphRB1) (Liu et al., 2011). Putative roles for RTK signaling in opioid tolerance were first shown in mice with global deletion of EphRB1 (Liu et al., 2011) as they failed to develop tolerance to spinal morphine administration. Similarly, we and others found that systemic or intrathecal co-administration of morphine alongside RTK inhibition via inhibitors of PDGFRβ (Wang et al., 2012; Li et al., 2020; Puig et al., 2020a), EGFR (Puig et al., 2020b), or VEGFR-2 (Lopez-Bellido et al., 2019) completely blocked tolerance. Together, these studies show that spinal RTK signaling is essential in morphine tolerance development. In addition, supraspinal inhibition of the RTK, FGFR, via intracerebroventricular (i.c.v.) injection also blocks tolerance to morphine injected subcutaneously (Fujita-Hamabe et al., 2011). Thus, other supraspinal structures of the pain circuitry may additionally contribute to RTK-mediated tolerance behaviors.

Precluding spinal signaling from one RTK at a time is sufficient to fully ablate tolerance. This apparent signaling redundancy raises the possibilities that: (1) spinal RTKs may work in parallel to transduce complex signaling cascades that specifically mediate tolerance and (2) that all signaling cascades recruited by RTKs are essential for tolerance. Interestingly, RTKs including EGFR and PDGFR-β were shown to heterodimerize *in vitro* (Habib et al., 1998; Saito et al., 2001). Heterodimerization could also happen *in vivo*, and co-transactivation of several spinal RTKs by MOR may be involved in mechanisms of tolerance. However, inhibition of RTKs individually alters tolerance in different ways depending on the RTK. For example, PDGFRβ inhibition only masks the expression of morphine tolerance (Wang et al., 2012), while EGFR inhibition completely blocks its development (Puig et al., 2020b). Importantly, these results have been reproduced by several independently conducted studies, highlighting the robustness of these findings (Wang et al., 2012; Li et al., 2020; Puig et al., 2020a).

In addition, we found that PDGFRβ inhibition blocks tolerance to several opioid analgesics used in the clinic including fentanyl, sufentanil, hydromorphone, and oxycodone (Puig et al., 2020a). Interestingly, these opioids have profoundly different pharmacokinetic and pharmacodynamic properties and have different signaling bias (Keith et al., 1998; Schmid et al., 2017). These findings show functional dissociation between MOR endocytosis, ligand signaling bias and tolerance, challenging the long-held hypotheses that mechanisms of MOR internalization (Whistler et al., 1999; Finn and Whistler, 2001) or of recruitment of β-arrestin2 (Bohn et al., 2000, 2004) are at the core of tolerance signaling. Instead, it suggests that PDGFRβ signaling could be a core mediator of opioid analgesic tolerance (Puig et al., 2020a). This is further supported by the fact that tolerance occurs independently of opioid-induced MOR internalization, and PDGFRβ inhibition does not modify levels of internalization while preventing tolerance (Puig et al., 2020a).

The precise RTK signaling pathways activated by opioid-stimulated MOR that mediate tolerance remain completely unknown. However, a recent *in vivo* study suggested that they could involve JNK signaling downstream of PDGFRβ (Li et al., 2020). Mechanisms of MOR-RTK transactivation in the spinal cord to mediate tolerance are similarly unclear. However, they seem to involve RTK ligand-dependent signaling pathways (Liu et al., 2011; Wang et al., 2012). Therefore, MOR may recruit RTK signaling in either an autocrine or a paracrine manner and RTKs may not necessarily need to be co-expressed with MOR. In addition, RTKs that have been involved in tolerance are closely phylogenetically related (Brunet et al., 2016). Indeed, VEGFR-2 and PDGFRβ share a direct ancestor gene, the RTK PDGF/VEGF receptor (Pvr) (Lopez-Bellido et al., 2019). This implies that involvement of RTKs in opioid tolerance could be a phylogenetically conserved function.

### Receptor tyrosine kinase signaling and reduced opioid analgesia to neuropathic pain

Neuropathic pain results from lesions or diseases of the somatosensory system that lead to a combination of inflammation and nerve compression (Dworkin et al., 2003, 2010). NP can also result from nerve damage as a consequence of prolonged chemotherapy (Murnion, 2018; Finnerup et al., 2021). Due to a lack of better alternatives, opioids are commonly used for NP (Quasthoff and Hartung, 2002; Chong and Bajwa, 2003; Lynch et al., 2004; Balayssac et al., 2009). Nevertheless, opioids are not very effective in treating NP as several clinical studies have shown that, despite high opioid dosage, NP could not be alleviated following opioid administration (Chaparro et al., 2012; Cooper et al., 2017). Although combination therapy between opioids and gabapentin, a blocker of voltage-gated calcium channels, proved effective, it has been associated with severely debilitating side-effects including nausea, constipation, and vomiting (Chaparro et al., 2012). Therefore, new therapeutic strategies and targets are needed for ameliorating NP.

NP can be modeled in rodents by inducing nerve injury on spinal nerves via spinal nerve ligation (SNL) (Kim and Chung, 1992) or chronic contraction injury (CCI) of the sciatic nerve (Bennett and Xie, 1988). In these models, low doses of opioids fail to produce analgesia. Several studies have established the impact of signaling from different RTKs in neuropathic pain development and maintenance in rodents including signaling by PDGFRα (Narita et al., 2005), FLT3 (Rivat et al., 2018), EphRB1 (Han et al., 2008), EGFR (Martin et al., 2017), or TrkA (Ugolini et al., 2007). In addition, a clinical study demonstrated that targeted inhibition of EGFR significantly reduces pain in male and female NP patients (Kersten et al., 2019). Groundbreaking recent discoveries also established direct involvement of RTK signaling in NP resistance to opioid analgesia. Pharmacological inhibition of either PDGFRβ (Donica et al., 2014) or EGFR (Puig et al., 2020b) restores analgesia to a dose of morphine previously ineffective on mechanical allodynia caused by SNL. This shows that PDGFRβ or EGFR inhibition is sufficient to restore morphine analgesic properties that were abolished by alterations caused by nerve injury. Importantly, administration of the same doses of PDGFRβ or EGFR inhibitors alone does not have any analgesic effect. In fact, we estimated that the dose of EGFR inhibitor used to restore morphine analgesia is ∼20-fold lower than the dose previously needed to induce analgesia (Nair and Jacob, 2016; Puig et al., 2020b). These results emphasize that RTK inhibitors restore morphine-mediated analgesia rather than causing analgesia by themselves. This indicates that recruitment of PDGFRβ and EGFR signaling by nerve injury activates signaling pathways that may block opioid analgesic signaling during NP. Moreover, these mechanisms resemble findings in the context of opioid tolerance (Wang et al., 2012; Puig et al., 2020a,b), and imply that convergent mechanisms between opioid tolerance and NP involve RTK signaling. Based on these observations, it was speculated that injured nerves release growth factors such as PDGF-B to activate PDGFRβ signaling and induce morphine-resistant states (Donica et al., 2014). It has also been proposed that this endogenous PDGF-B release by injured nerves, is similar to the release of PDGF-B in response to opioid administration, leading to activation of MOR to mediate tolerance (Wang et al., 2012). In conclusion, RTK signaling mediating opioid analgesic resistance may form a mechanistic link between neuropathic pain development and opioid tolerance (Mao et al., 1995; Mayer et al., 1999; Joseph et al., 2010; Donica et al., 2014; Puig et al., 2020b).

### Receptor tyrosine kinase signaling and opioid dependence

There is a complex bidirectional relationship between RTK gene expression and opioid dependence. Dorval and colleagues showed that mice that overexpress FGF21, an FGFR ligand (FGF21-Tg mice, 50-fold overexpression), have a reduced preference to morphine in a conditioned place preference paradigm (Dorval et al., 2022). Further, naloxone-precipitated physical dependence behavior, (i.e., number of vertical jumps post-naloxone injection) is depressed in FGF21-Tg mice compared to wildtype littermates, suggesting that acute morphine physical dependence is regulated by FGF21 activity. Interestingly, morphine analgesia and tolerance development were not altered in FGF21-Tg mice, showing that FGF21 plays a role in opioid dependence but not in analgesia or tolerance. These findings are consistent with previous studies showing that oxycodone self-administration is associated with elevated striatal *fgf2*, *fgfr2*, and *fgfr3*mRNA levels during incubation of oxycodone seeking (Blackwood et al., 2019). Furthermore, these changes in FGF receptor gene expression are associated with elevated *c-fos* mRNA expression in the dorsal striatum, and elevated *junB* mRNA levels in these same regions. Given that the striatum is an important region of the reward circuitry, Blackwood and collaborators (Blackwood et al., 2019) hypothesized that incubation of oxycodone seeking, a behavior correlated with future dependence, is mediated at least in-part by FGF2-dependent signaling. However, the mechanisms of FGF receptor-driven opioid dependence remain unknown.

Other studies have also indicated that brain-derived neurotrophic factor (BDNF), as well as its receptor, tropomyosin receptor kinase B (TrkB), may also play integral roles in opioid dependence and withdrawal development. BDNF, TrkB, IGF1, and IFG1R mRNA levels were found to be elevated in rodents frontal cortex in a model of physical dependence to morphine. In addition, BDNF was upregulated in hippocampus and midbrain (Peregud et al., 2016). Recruitment of BDNF-TrkB signaling by MOR during exposure to opioids or during withdrawal may be mediated via mechanisms of atypical GPCR-RTK transactivation involving a ROS, nitric oxide (NO) (as illustrated in Figure 3C). Withdrawal associated elevation of BDNF and TrkB and their respective receptors is markedly lower in animals pretreated with the nitric oxide synthase (NOS) inhibitor L-N^G^-nitroarginine methyl ester (L-NAME). L-NAME-treated animals also exhibited depressed amounts of phosphorylated TrkB following abstinence from morphine (Peregud et al., 2016). Confirming the role of TrkB signaling in withdrawal behaviors, a recent study showed that rats pre-treated with ANA-12, a TrkB antagonist, displayed greater drug dependence and significantly more spontaneous withdrawal behaviors after a chronic treatment with morphine. Furthermore, BDNF levels in the cerebrospinal fluid (CSF) of ANA-12 treated animals are depressed during morphine dependence, and elevated during withdrawal (Rezamohammadi et al., 2020). Together these studies show that FGFR and TrkB activation may have protective effects against physical dependence, highlighting that this should be carefully considered in the process of testing RTK targeting therapies to treat opioid side-effects.

One hallmark of chronic opioid use which occurs upon opioid withdrawal is opioid-induced hyperalgesia (OIH). Of interest, Ephrin receptors, the most prominent subfamily of RTKs, which are commonly associated with neuron-neuron and neuron-glia interactions have been implicated in OIH. In a rat model of remifentanil-induced hyperalgesia, remifentanil-induced decrease of mechanical and thermal pain threshold has been correlated with elevated spinal Fos protein levels. Interestingly, these effects were reversed by inhibition of either EphB ligand (via EphB1-Fc) or the NMDA receptor (NMDAR) (via MK801) (Xia et al., 2014). Further, intrathecal injection of ephrinB/EphB agonist, was sufficient to induce significant hyperalgesia in a NMDAR-dependent manner (Xia et al., 2014), showing that activation of ephrinB/EphB pathways are sufficient to mediate OIH development via NMDAR (Xia et al., 2014). Importantly, other RTKs are also known to be involved in NMDAR-mediated OIH, including BDNF-TrkB signaling in the spinal dorsal horn. Notably, previous work demonstrated that morphine-induced hyperalgesia occurs because of MOR-dependent BDNF release leading to a downregulation of K^+^/Cl^–^ co-transporter (KCC2) in rat spinal lamina neurons (Ferrini et al., 2013). The resulting Cl^–^ dysequilibrium serves as a driver of hyperalgesia which is reversible by inhibition of BDNF-TrkB *or* via prevention of KCC2 downregulation (Ferrini et al., 2017). Further, this reversible anion transport dysfunction induces a dampening of GABAergic and glycinergic spinal signaling and elevated NMDAR activity (Li et al., 2016).

### Receptor tyrosine kinase signaling and opioid reward

RTK signaling and opioid reward involve midbrain dopamine neurons in the ventral tegmental area (VTA) which project to the nucleus accumbens (NAc) in the striatum. In the context of opioids, morphine promotes activation of striatal D_1_ receptor (D1R)-expressing MSNs which increase reward behaviors and decreases dopamine D_2_ receptor-expressing (D2R) MSNs which promote aversion. TrkB is expressed in both D1R^+^ and D2R^+^ MSNs (Freeman et al., 2003; Baydyuk et al., 2011) and most evidence about involvement of RTK signaling in opioid reward derives from work analyzing TrkB and morphine administration. Indeed, there is decreased conditioned place preference (CPP) for morphine when the selective TrkB antagonist, ANA-12, is injected into the NAc of rats (Jorjani et al., 2021). However, it was also shown that selective knockout of TrkB from D1R^+^ MSNs of the NAc in mice, enhances morphine CPP while knockout of D2R^+^ MSNs produces no change (Koo et al., 2014). Moreover, knocking out TrkB in the VTA produces a similar effect as TrkB knockout in D1R^+^ MSNs in the NAc with enhanced morphine CPP (Koo et al., 2014). Overall, these findings suggest that TrkB-based RTK signaling in D1R^+^ versus D2R^+^ MSNs mediates opposing actions that together modulate opioid-induced behaviors and that these actions are dependent on striatal dopamine release from projections of midbrain dopaminergic neurons.

IR signaling has also been implicated in opioid reward. In the hippocampus and hypothalamus, morphine induces IR phosphorylation in wildtype, but not MOR knockout mice, suggesting that MORs are able to transactivate IRs in these structures (Li et al., 2003). Additionally, given the important role of glutamatergic neurotransmission in drug reward (Britt et al., 2012), the increases in presynaptic glutamate release in the NAc in response to IR activation (Fetterly et al., 2021) may provide a further RTK-mediated mechanism for opioid actions. In contrast, insulin growth factor like receptor (IGFR) activation decreases presynaptic glutamate release in the same neuronal population, demonstrating differential effects depending on the RTK. Additional involvement of IR in opioid reward is supported by work showing that prolonged morphine-activated MORs *in vitro* can cause desensitization of IR signaling to Akt and ERK cascades (Li et al., 2003), both of which have been implicated in reward (Shi et al., 2014; Zamora-Martinez and Edwards, 2014). Inhibition of ERK in the NAc shell prevents development of morphine CPP (Xu et al., 2012). While, in a separate study, Russo and colleagues showed that downregulation of Akt and IR subunit 2, an essential component of functional IR signaling, in the VTA results in reward tolerance as shown by decreased CPP behaviors over time (Russo et al., 2007). Together, these results further reinforce the involvement of IRs in modulation of MOR-mediated reward signaling within the NAc. Further studies to establish if this could be generalized to other opioids remain necessary. For example, PDGFRβ is also expressed in brain regions involved in reward and addiction such as mPFC, NAc and dStr (Balayssac et al., 2009; Bor et al., 2017) and PDGFRβ levels were shown to be altered in the striatum and midbrain of rodents with disrupted dopaminergic signaling, a central component for reward signaling (Masuo et al., 2004). Overall, this work suggests RTKs could be promising, yet understudied, candidates to mitigate morphine reward, especially IRs.

## Clinical implications

Treating human disease with RTK-targeted therapies is an established standard of care as a therapeutic strategy for cancer. Indeed, RTK inhibitors serve as the gold standard treatment of malignancies (Savage and Antman, 2002; Elisei et al., 2013; Sim et al., 2018). These medications are being explored for putative efficacy in other, non-oncological conditions. Specifically, EGFR and PDGFR inhibitors have recently received attention for their clinical therapeutic potential against pain. Several case reports have described analgesic effects by EGFR inhibitors in patients with severe pain (Kersten and Cameron, 2012; Kersten et al., 2015). Patients with either cancer pain (Moryl et al., 2006; Macey et al., 2009) or different types of neuropathic pain (Kersten et al., 2015) were treated with EGFR inhibitors which significantly improved their pain score after a few days. Most interestingly, in a clinical study led by Kersten and collaborators (Kersten et al., 2013), half the patients who experienced immediate pain relief following administration of the EGFR inhibitor cetuximab also decreased their required opioid doses. The authors concluded that cetuximab reversed opioid tolerance. Similarly, PDGFR-β inhibitor imatinib induced analgesia in cancer patients (Stankovic Stojanovic et al., 2011; Kutlar, 2013). Based on the promise of these recent clinical studies and case reports, it is possible that the improved pain relief observed with RTK inhibitors is due to the reversal of pre-existing opioid tolerance. We propose that combined treatment with opioids and RTK inhibitors may decouple the intertwined pathways mediating analgesia and tolerance.

## Conclusion

Uncoupling analgesia from undesirable effects of opioids by RTK inhibitors could therefore enable patients to maintain opioid efficacy at smaller doses, mitigating the risk of side-effects associated with chronic opioid use. Importantly, in rodents, efficacious doses to mitigate opioid tolerance appear to be significantly lower than those required to treat cancers. This holds the promise that RTK doses required for effective prevention of opioid side-effects in humans should not have a deleterious impact that could outweigh advantages of RTK inhibitors. Nevertheless, more work is clearly needed to better understand how RTK inhibitors work in the context of tolerance. It is still unknown if RTK inhibitors could be used to treat opioid withdrawal symptoms or prevent rewarding properties of opioids in humans. It is imperative that future studies assess the power of concurrent opioid-RTK inhibitors treatments both in the clinic and in pre-clinical models of pain. If successful, RTK inhibitors may represent a promising new class of drugs to treat pain more safely in conjunction with opioids and therefore positively impact the lives of millions living with chronic pain.

## Author contributions

MG, NS, and BW contributed equally by generating a first draft of this manuscript. LP, ZF, and RL contributed to the writing of this manuscript. SP initiated the manuscript, supervised collection of cited references, and contributed to first and final drafts. All authors contributed to the article and approved the submitted version.

## References

[B1] Adhikary S. Williams J. T. (2022). Cellular tolerance induced by chronic opioids in the central nervous system. *Front. Syst. Neurosci.* 16:937126. 10.3389/fnsys.2022.937126 35837149 PMC9273719

[B2] Algera M. H. Kamp J. Van Der Schrier R. Van Velzen M. Niesters M. Aarts L. (2019). Opioid-induced respiratory depression in humans: A review of pharmacokinetic-pharmacodynamic modelling of reversal. *Br. J. Anaesth.* 122 e168–e179. 10.1016/j.bja.2018.12.023 30915997

[B3] Al-Hasani R. Bruchas M. R. (2011). Molecular mechanisms of opioid receptor-dependent signaling and behavior. *Anesthesiology* 115 1363–1381. 10.1097/ALN.0b013e318238bba6 22020140 PMC3698859

[B4] Allouche S. Noble F. Marie N. (2014). Opioid receptor desensitization: Mechanisms and its link to tolerance. *Front. Pharmacol.* 5:280. 10.3389/fphar.2014.00280 25566076 PMC4270172

[B5] Alvarez V. A. Arttamangkul S. Dang V. Salem A. Whistler J. L. Von Zastrow M. (2002). Mu-opioid receptors: Ligand-dependent activation of potassium conductance, desensitization, and internalization. *J. Neurosci.* 22 5769–5776. 10.1523/JNEUROSCI.22-13-05769.2002 12097530 PMC6758217

[B6] Azolosa J. L. Stitzer M. L. Greenwald M. K. (1994). Opioid physical dependence development: Effects of single versus repeated morphine pretreatments and of subjects’ opioid exposure history. *Psychopharmacology (Berl)* 114 71–80. 10.1007/BF02245446 7846209

[B7] Balayssac D. Cayre A. Ling B. Maublant J. Penault-Llorca F. Eschalier A. (2009). Increase in morphine antinociceptive activity by a P-glycoprotein inhibitor in cisplatin-induced neuropathy. *Neurosci. Lett.* 465 108–112. 10.1016/j.neulet.2009.09.003 19733628

[B8] Baltensperger K. Karoor V. Paul H. Ruoho A. Czech M. P. Malbon C. C. (1996). The β-adrenergic receptor is a substrate for the insulin receptor tyrosine kinase. *J. Biol. Chem.* 271 1061–1064. 10.1074/jbc.271.2.1061 8557631

[B9] Basbaum A. I. Bautista D. M. Scherrer G. Julius D. (2009). Cellular and molecular mechanisms of pain. *Cell* 139 267–284. 10.1016/j.cell.2009.09.028 19837031 PMC2852643

[B10] Baydyuk M. Russell T. Liao G. Y. Zang K. An J. J. Reichardt L. F. (2011). TrkB receptor controls striatal formation by regulating the number of newborn striatal neurons. *Proc. Natl. Acad. Sci. U.S.A.* 108 1669–1674. 10.1073/pnas.1004744108 21205893 PMC3029684

[B11] Belcheva M. M. Clark A. L. Haas P. D. Serna J. S. Hahn J. W. Kiss A. (2005). Mu and kappa opioid receptors activate ERK/MAPK via different protein kinase C isoforms and secondary messengers in astrocytes. *J. Biol. Chem.* 280 27662–27669. 10.1074/jbc.M502593200 15944153 PMC1400585

[B12] Belcheva M. M. Szucs M. Wang D. Sadee W. Coscia C. J. (2001). Mu-opioid receptor-mediated ERK activation involves calmodulin-dependent epidermal growth factor receptor transactivation. *J. Biol. Chem.* 276 33847–33853. 10.1074/jbc.M101535200 11457825

[B13] Belcheva M. M. Tan Y. Heaton V. M. Clark A. L. Coscia C. J. (2003). Mu opioid transactivation and down-regulation of the epidermal growth factor receptor in astrocytes: Implications for mitogen-activated protein kinase signaling. *Mol. Pharmacol.* 64 1391–1401. 10.1124/mol.64.6.1391 14645669

[B14] Bennett G. J. Xie Y.-K. (1988). A peripheral mononeuropathy in rat that produces disorders of pain sensation like those seen in man. *Pain* 33 87–107. 10.1016/0304-3959(88)90209-6 2837713

[B15] Benyamin R. Trescot A. M. Datta S. Buenaventura R. Adlaka R. Sehgal N. (2008). Opioid complications and side effects. *Pain Physician* 11 S105–S120. 10.36076/ppj.2008/11/S105 18443635

[B16] Blackwood C. A. Leary M. Salisbury A. Mccoy M. T. Cadet J. L. (2019). Escalated oxycodone self-administration causes differential striatal mRNA expression of FGFs and IEGs following abstinence-associated incubation of oxycodone craving. *Neuroscience* 415 173–183. 10.1016/j.neuroscience.2019.07.030 31351142 PMC7448685

[B17] Bohn L. M. Dykstra L. A. Lefkowitz R. J. Caron M. G. Barak L. S. (2004). Relative opioid efficacy is determined by the complements of the G protein-coupled receptor desensitization machinery. *Mol. Pharmacol.* 66 106–112. 10.1124/mol.66.1.106 15213301

[B18] Bohn L. M. Gainetdinov R. R. Lin F. T. Lefkowitz R. J. Caron M. G. (2000). Mu-opioid receptor desensitization by beta-arrestin-2 determines morphine tolerance but not dependence. *Nature* 408 720–723. 10.1038/35047086 11130073

[B19] Bohn L. M. Gainetdinov R. R. Sotnikova T. D. Medvedev I. O. Lefkowitz R. J. Dykstra L. A. (2003). Enhanced rewarding properties of morphine, but not cocaine, in beta(arrestin)-2 knock-out mice. *J. Neurosci.* 23 10265–10273. 10.1523/JNEUROSCI.23-32-10265.2003 14614085 PMC6741024

[B20] Bohn L. M. Lefkowitz R. J. Caron M. G. (2002). Differential mechanisms of morphine antinociceptive tolerance revealed in (beta)arrestin-2 knock-out mice. *J. Neurosci.* 22 10494–10500. 10.1523/JNEUROSCI.22-23-10494.2002 12451149 PMC6758751

[B21] Bohn L. M. Lefkowitz R. J. Gainetdinov R. R. Peppel K. Caron M. G. Lin F. T. (1999). Enhanced morphine analgesia in mice lacking beta-arrestin 2. *Science* 286 2495–2498. 10.1126/science.286.5449.2495 10617462

[B22] Bor A. Nishijo M. Nishimaru H. Nakamura T. Tran N. N. Van Le Q. (2017). Effects of high fat diet and perinatal dioxin exposure on development of body size and expression of platelet-derived growth factor receptor beta in the rat brain. *J. Integr. Neurosci.* 16 453–470. 10.3233/JIN-170025 28891521

[B23] Britt J. P. Benaliouad F. Mcdevitt R. A. Stuber G. D. Wise R. A. Bonci A. (2012). Synaptic and behavioral profile of multiple glutamatergic inputs to the nucleus accumbens. *Neuron* 76 790–803. 10.1016/j.neuron.2012.09.040 23177963 PMC3607383

[B24] Brunet F. G. Volff J. N. Schartl M. (2016). Whole genome duplications shaped the receptor tyrosine kinase repertoire of jawed vertebrates. *Genome Biol. Evol.* 8 1600–1613. 10.1093/gbe/evw103 27260203 PMC4898815

[B25] Burma N. E. Kwok C. H. Trang T. (2017). Therapies and mechanisms of opioid withdrawal. *Pain Manag.* 7 455–459. 10.2217/pmt-2017-0028 29125396

[B26] Cattaneo F. Guerra G. Parisi M. De Marinis M. Tafuri D. Cinelli M. (2014). Cell-surface receptors transactivation mediated by g protein-coupled receptors. *Int. J. Mol. Sci.* 15 19700–19728. 10.3390/ijms151119700 25356505 PMC4264134

[B27] Chakrabarti S. Gintzler A. R. (2007). Phosphorylation of Gαs influences its association with the μ-opioid receptor and is modulated by long-term morphine exposure. *Mol. Pharmacol.* 72 753–760. 10.1124/mol.107.036145 17576791

[B28] Chakrabarti S. Chang A. Gintzler A. R. (2010). Subcellular localization of μ-opioid receptor GS signaling. *J. Pharmacol. Exp. Therap.* 333 193–200. 10.1124/jpet.109.165142 20097777 PMC2846030

[B29] Chakrabarti S. Regec A. Gintzler A. R. (2005). Biochemical demonstration of mu-opioid receptor association with Gsα: Enhancement following morphine exposure. *Mol. Brain Res.* 135 217–224. 10.1016/j.molbrainres.2004.12.016 15857684

[B30] Chaparro L. E. Wiffen P. J. Moore R. A. Gilron I. (2012). Combination pharmacotherapy for the treatment of neuropathic pain in adults. *Cochrane Database Syst. Rev.* 12:CD008943. 10.1002/14651858.CD008943.pub2 22786518 PMC6481651

[B31] Chaturvedi K. Bandari P. Chinen N. Howells R. D. (2001). Proteasome involvement in agonist-induced down-regulation of μ and δ opioid receptors. *J. Biol. Chem.* 276 12345–12355. 10.1074/jbc.M008054200 11152677

[B32] Chen Y. Geis C. Sommer C. (2008a). Activation of TRPV1 contributes to morphine tolerance: Involvement of the mitogen-activated protein kinase signaling pathway. *J. Neurosci.* 28 5836–5845. 10.1523/JNEUROSCI.4170-07.2008 18509045 PMC6670790

[B33] Chen Y. Long H. Wu Z. Jiang X. Ma L. (2008b). EGF transregulates opioid receptors through Egfr-mediated Grk2 phosphorylation and activation. *Mol. Biol. Cell* 19 2973–2983. 10.1091/mbc.e07-10-1058 18463167 PMC2441682

[B34] Chieng B. Christie M. (1994). Hyperpolarization by opioids acting on μ−receptors of a sub-population of rat periaqueductal gray neurones in vitro. *Br. J. Pharmacol.* 113 121–128. 10.1111/j.1476-5381.1994.tb16183.x 7812601 PMC1510059

[B35] Chong M. S. Bajwa Z. H. (2003). Diagnosis and treatment of neuropathic pain. *J. Pain Symptom Manag.* 25 S4–S11. 10.1016/S0885-3924(03)00064-2 12694987

[B36] Choudhary C. Olsen J. V. Brandts C. Cox J. Reddy P. N. Böhmer F. D. (2009). Mislocalized activation of oncogenic RTKs switches downstream signaling outcomes. *Mol. Cell* 36 326–339. 10.1016/j.molcel.2009.09.019 19854140

[B37] Clayton C. C. Bruchas M. R. Lee M. L. Chavkin C. (2010). Phosphorylation of the mu-opioid receptor at tyrosine 166 (Tyr3.51) in the DRY motif reduces agonist efficacy. *Mol. Pharmacol.* 77 339–347. 10.1124/mol.109.060558 19959593 PMC2835424

[B38] Collett B. J. (1998). Opioid tolerance: The clinical perspective. *Br. J. Anaesth.* 81 58–68. 10.1093/bja/81.1.58 9771273

[B39] Conibear A. E. Kelly E. (2019). A biased view of μ-opioid receptors? *Mol. Pharmacol.* 96 542–549. 10.1124/mol.119.115956 31175184 PMC6784500

[B40] Connor M. Christie M. J. (1999). Opioid receptor signalling mechanisms. *Clin. Exp. Pharmacol. Physiol.* 26 493–499. 10.1046/j.1440-1681.1999.03049.x 10405772

[B41] Cooper T. E. Chen J. Wiffen P. J. Derry S. Carr D. B. Aldington D. (2017). Morphine for chronic neuropathic pain in adults. *Cochrane Database Syst. Rev.* 5:CD011669. 10.1002/14651858.CD011669.pub2 28530786 PMC6481499

[B42] Corder G. Tawfik V. L. Wang D. Sypek E. I. Low S. A. Dickinson J. R. (2017). Loss of mu opioid receptor signaling in nociceptors, but not microglia, abrogates morphine tolerance without disrupting analgesia. *Nat. Med.* 23 164–173. 10.1038/nm.4262 28092666 PMC5296291

[B43] Dalle S. Ricketts W. Imamura T. Vollenweider P. Olefsky J. M. (2001). Insulin and insulin-like growth factor I receptors utilize different G protein signaling components. *J. Biol. Chem.* 276 15688–15695. 10.1074/jbc.M010884200 11278773

[B44] Daub H. Ulrich Weiss F. Wallasch C. Ullrich A. (1996). Role of transactivation of the EGF receptor in signalling by G-protein-coupled receptors. *Nature* 379 557–560. 10.1038/379557a0 8596637

[B45] Delcourt N. Bockaert J. Marin P. (2007). Gpcr-jacking: From a new route in RTK signalling to a new concept in GPCR activation. *Trends Pharmacol. Sci.* 28 602–607. 10.1016/j.tips.2007.09.007 18001849

[B46] Di Liberto V. Mudò G. Belluardo N. (2019). Crosstalk between receptor tyrosine kinases (Rtks) and G protein-coupled receptors (GPCR) in the brain: Focus on heteroreceptor complexes and related functional neurotrophic effects. *Neuropharmacology* 152 67–77. 10.1016/j.neuropharm.2018.11.018 30445101

[B47] Doll C. Konietzko J. Pöll F. Koch T. Höllt V. Schulz S. (2011). Agonist-selective patterns of μ-opioid receptor phosphorylation revealed by phosphosite-specific antibodies. *Br. J. Pharmacol.* 164 298–307. 10.1111/j.1476-5381.2011.01382.x 21449911 PMC3174411

[B48] Donica C. L. Cui Y. Shi S. Gutstein H. B. (2014). Platelet-derived growth factor receptor-beta antagonism restores morphine analgesic potency against neuropathic pain. *PLoS One* 9:e97105. 10.1371/journal.pone.0097105 24820332 PMC4018247

[B49] Doronin S. Shumay E. Wang H.-Y. Malbon C. C. (2002). Akt mediates sequestration of the β2-adrenergic receptor in response to insulin. *J. Biol. Chem.* 277 15124–15131. 10.1074/jbc.M108771200 11809767

[B50] Dorval L. Knapp B. I. Majekodunmi O. A. Eliseeva S. Bidlack J. M. (2022). Mice with high Fgf21 serum levels had a reduced preference for morphine and an attenuated development of acute antinociceptive tolerance and physical dependence. *Neuropharmacology* 202:108858. 10.1016/j.neuropharm.2021.108858 34715121 PMC8627472

[B51] Doyle T. Esposito E. Bryant L. Cuzzocrea S. Salvemini D. (2013). Nadph-oxidase 2 activation promotes opioid-induced antinociceptive tolerance in mice. *Neuroscience* 241 1–9. 10.1016/j.neuroscience.2013.02.042 23454539 PMC4184413

[B52] Du Z. Lovly C. M. (2018). Mechanisms of receptor tyrosine kinase activation in cancer. *Mol. Cancer* 17:58. 10.1186/s12943-018-0782-4 29455648 PMC5817791

[B53] Duttaroy A. Yoburn B. C. (1995). The effect of intrinsic efficacy on opioid tolerance. *Anesthesiology* 82 1226–1236. 10.1097/00000542-199505000-00018 7741298

[B54] Dworkin R. H. Backonja M. Rowbotham M. C. Allen R. R. Argoff C. R. Bennett G. J. (2003). Advances in neuropathic pain: Diagnosis, mechanisms, and treatment recommendations. *Arch. Neurol.* 60 1524–1534. 10.1001/archneur.60.11.1524 14623723

[B55] Dworkin R. H. O’connor A. B. Audette J. Baron R. Gourlay G. K. Haanpää M. L. (2010). Recommendations for the pharmacological management of neuropathic pain: An overview and literature update. *Mayo Clin. Proc.* 85 S3–S14. 10.4065/mcp.2009.0649 20194146 PMC2844007

[B56] Eccleston P. A. Funa K. Heldin C. H. (1993). Expression of platelet-derived growth factor (PDGF) and PDGF alpha- and beta-receptors in the peripheral nervous system: An analysis of sciatic nerve and dorsal root ganglia. *Dev. Biol.* 155 459–470. 10.1006/dbio.1993.1044 8432400

[B57] Elisei R. Schlumberger M. J. Muller S. P. Schoffski P. Brose M. S. Shah M. H. (2013). Cabozantinib in progressive medullary thyroid cancer. *J. Clin. Oncol.* 31 3639–3646. 10.1200/JCO.2012.48.4659 24002501 PMC4164813

[B58] Epstein D. H. Preston K. L. Jasinski D. R. (2006). Abuse liability, behavioral pharmacology, and physical-dependence potential of opioids in humans and laboratory animals: Lessons from tramadol. *Biol. Psychol.* 73 90–99. 10.1016/j.biopsycho.2006.01.010 16497429 PMC2943845

[B59] Favelyukis S. Till J. H. Hubbard S. R. Miller W. T. (2001). Structure and autoregulation of the insulin-like growth factor 1 receptor kinase. *Nat. Struct. Biol.* 8 1058–1063. 10.1038/nsb721 11694888

[B60] Ferrini F. Lorenzo L. E. Godin A. G. Quang M. L. De Koninck Y. (2017). Enhancing Kcc2 function counteracts morphine-induced hyperalgesia. *Sci. Rep.* 7:3870. 10.1038/s41598-017-04209-3 28634406 PMC5478677

[B61] Ferrini F. Trang T. Mattioli T. A. Laffray S. Del’guidice T. Lorenzo L. E. (2013). Morphine hyperalgesia gated through microglia-mediated disruption of neuronal Cl(–) homeostasis. *Nat. Neurosci.* 16 183–192. 10.1038/nn.3295 23292683 PMC4974077

[B62] Fetterly T. L. Oginsky M. F. Nieto A. M. Alonso-Caraballo Y. Santana-Rodriguez Z. Ferrario C. R. (2021). Insulin bidirectionally alters NAc glutamatergic transmission: Interactions between insulin receptor activation, endogenous opioids, and glutamate release. *J. Neurosci.* 41 2360–2372. 10.1523/JNEUROSCI.3216-18.2021 33514676 PMC7984597

[B63] Finn A. K. Whistler J. L. (2001). Endocytosis of the mu opioid receptor reduces tolerance and a cellular hallmark of opiate withdrawal. *Neuron* 32 829–839. 10.1016/S0896-6273(01)00517-7 11738029

[B64] Finnerup N. B. Kuner R. Jensen T. S. (2021). Neuropathic pain: From mechanisms to treatment. *Physiol. Rev.* 101 259–301. 10.1152/physrev.00045.2019 32584191

[B65] Franz B. Cronin C. E. Pagan J. A. (2021). What strategies are hospitals adopting to address the opioid epidemic? Evidence from a national sample of nonprofit hospitals. *Public Health Rep.* 136 228–238. 10.1177/0033354920968805 33176117 PMC8093846

[B66] Freeman A. Y. Soghomonian J. J. Pierce R. C. (2003). Tyrosine kinase B and C receptors in the neostriatum and nucleus accumbens are co-localized in enkephalin-positive and enkephalin-negative neuronal profiles and their expression is influenced by cocaine. *Neuroscience* 117 147–156. 10.1016/S0306-4522(02)00802-3 12605901

[B67] Fujita-Hamabe W. Nakamoto K. Tokuyama S. (2011). Involvement of NCAM and FGF receptor signaling in the development of analgesic tolerance to morphine. *Eur. J. Pharmacol.* 672 77–82. 10.1016/j.ejphar.2011.04.029 21549695

[B68] García-Sáinz J. A. Romero-Ávila M. T. Del Carmen Medina L. (2010). Dissecting how receptor tyrosine kinases modulate G protein-coupled receptor function. *Eur. J. Pharmacol.* 648 1–5. 10.1016/j.ejphar.2010.08.049 20828551

[B69] Gavi S. Yin D. Shumay E. Wang H.-Y. Malbon C. C. (2007). Insulin-like growth factor-I provokes functional antagonism and internalization of β1-adrenergic receptors. *Endocrinology* 148 2653–2662. 10.1210/en.2006-1569 17363461

[B70] Gialeli C. Nikitovic D. Kletsas D. Theocharis A. D. Tzanakakis G. N. Karamanos N. K. (2014). PDGF/PDGFR signaling and targeting in cancer growth and progression: Focus on tumor microenvironment and cancer-associated fibroblasts. *Curr. Pharm. Des.* 20 2843–2848. 10.2174/13816128113199990592 23944365

[B71] Gillis A. Gondin A. B. Kliewer A. Sanchez J. Lim H. D. Alamein C. (2020). Low intrinsic efficacy for G protein activation can explain the improved side effect profiles of new opioid agonists. *Sci. Signal.* 13:eaaz3140. 10.1126/scisignal.aaz3140 32234959

[B72] Gregus A. M. Inra C. N. Giordano T. P. III Costa A. C. S. Rajadhyaksha A. M. Inturrisi C. E. (2010). Spinal mediators that may contribute selectively to antinociceptive tolerance but not other effects of morphine as revealed by deletion of GluR5. *Neuroscience* 169 475–487. 10.1016/j.neuroscience.2010.03.051 20359526 PMC2900492

[B73] Habib A. A. Hognason T. Ren J. Stefansson K. Ratan R. R. (1998). The epidermal growth factor receptor associates with and recruits phosphatidylinositol 3-kinase to the platelet-derived growth factor beta receptor. *J. Biol. Chem.* 273 6885–6891. 10.1074/jbc.273.12.6885 9506992

[B74] Haglund K. Rusten T. E. Stenmark H. (2007). Aberrant receptor signaling and trafficking as mechanisms in oncogenesis. *Crit. Rev. Oncog.* 13 39–74. 10.1615/CritRevOncog.v13.i1.20 17956217

[B75] Haglund K. Sigismund S. Polo S. Szymkiewicz I. Di Fiore P. P. Dikic I. (2003). Multiple monoubiquitination of Rtks is sufficient for their endocytosis and degradation. *Nat. Cell Biol.* 5 461–466. 10.1038/ncb983 12717448

[B76] Han Y. Song X.-S. Liu W.-T. Henkemeyer M. Song X.-J. (2008). Targeted mutation of EphB1 receptor prevents development of neuropathic hyperalgesia and physical dependence on morphine in mice. *Mol. Pain* 4:60. 10.1186/1744-8069-4-60 19025592 PMC2605438

[B77] Hayhurst C. J. Durieux M. E. (2016). Differential opioid tolerance and opioid-induced hyperalgesia: A clinical reality. *Anesthesiology* 124 483–488. 10.1097/ALN.0000000000000963 26594912

[B78] Heckman C. A. Wade J. G. (2018). Protein kinase C: Its role in RTK processing. *Trends Cancer Res.* 13 1–28.

[B79] Henry S. G. Wilsey B. L. Melnikow J. Iosif A. M. (2015). Dose escalation during the first year of long-term opioid therapy for chronic pain. *Pain Med.* 16 733–744. 10.1111/pme.12634 25529548 PMC4390410

[B80] Herrera J. J. Nesic O. Narayana P. A. (2009). Reduced vascular endothelial growth factor expression in contusive spinal cord injury. *J. Neurotrauma.* 26 995–1003. 10.1089/neu.2008.0779 19257807 PMC2848947

[B81] Hill R. Disney A. Conibear A. Sutcliffe K. Dewey W. Husbands S. (2018). The novel μ−opioid receptor agonist PZM21 depresses respiration and induces tolerance to antinociception. *Br. J. Pharmacol.* 175 2653–2661. 10.1111/bph.14224 29582414 PMC6003631

[B82] Holland K. M. Depadilla L. Gervin D. W. Parker E. M. Wright M. (2021). The evolution of the overdose epidemic and CDC’s research response: A commentary. *Drugs Context* 10. 10.7573/dic.2021-8-2 34970319 PMC8687091

[B83] Honegger A. Kris R. Ullrich A. Schlessinger J. (1989). Evidence that autophosphorylation of solubilized receptors for epidermal growth factor is mediated by intermolecular cross-phosphorylation. *Proc. Natl Acad. Sci. U.S.A.* 86 925–929. 10.1073/pnas.86.3.925 2915986 PMC286591

[B84] Huerta J. J. Diaz-Trelles R. Naves F. J. Llamosas M. M. Del Valle M. E. Vega J. A. (1996). Epidermal growth factor receptor in adult human dorsal root ganglia. *Anat. Embryol. (Berl)* 194 253–257. 10.1007/BF00187136 8849672

[B85] Hupfeld C. J. Olefsky J. M. (2007). Regulation of receptor tyrosine kinase signaling by GRKs and β-arrestins. *Annu. Rev. Physiol.* 69 561–577. 10.1146/annurev.physiol.69.022405.154626 17002595

[B86] Jean-Charles P.-Y. Kaur S. Shenoy S. K. (2017). GPCR signaling via β-arrestin-dependent mechanisms. *J. Cardiovasc. Pharmacol.* 70:142. 10.1097/FJC.0000000000000482 28328745 PMC5591062

[B87] Jorjani H. Joneidi M. Vafaei A. A. Rashidy-Pour A. Sameni H. Bandegi A. R. (2021). Microinjection of the BDNF receptor antagonist ANA-12 into the nucleus accumbens and medial-prefrontal cortex attenuates morphine-induced reward memory, and alterations of BDNF levels and apoptotic cells in rats. *Pharmacol. Biochem. Behav.* 201:173111. 10.1016/j.pbb.2021.173111 33444602

[B88] Joseph E. K. Reichling D. B. Levine J. D. (2010). Shared mechanisms for opioid tolerance and a transition to chronic pain. *J. Neurosci.* 30 4660–4666. 10.1523/JNEUROSCI.5530-09.2010 20357116 PMC2857996

[B89] Karaman M. W. Herrgard S. Treiber D. K. Gallant P. Atteridge C. E. Campbell B. T. (2008). A quantitative analysis of kinase inhibitor selectivity. *Nat Biotechnol* 26 127–132. 10.1038/nbt1358 18183025

[B90] Keith D. E. Anton B. Murray S. R. Zaki P. A. Chu P. C. Lissin D. V. (1998). mu-Opioid receptor internalization: Opiate drugs have differential effects on a conserved endocytic mechanism in vitro and in the mammalian brain. *Mol. Pharmacol.* 53 377–384. 10.1124/mol.53.3.377 9495801

[B91] Keith D. E. Murray S. R. Zaki P. A. Chu P. C. Lissin D. V. Kang L. (1996). Morphine activates opioid receptors without causing their rapid internalization. *J. Biol. Chem.* 271 19021–19024. 10.1074/jbc.271.32.19021 8702570

[B92] Kelly E. Bailey C. P. Henderson G. (2008). Agonist-selective mechanisms of Gpcr desensitization. *Br. J. Pharmacol.* 153 S379–S388. 10.1038/sj.bjp.0707604 18059321 PMC2268061

[B93] Kenakin T. (2011). Functional selectivity and biased receptor signaling. *J. Pharmacol. Exp. Ther.* 336 296–302. 10.1124/jpet.110.173948 21030484

[B94] Kersten C. Cameron M. G. (2012). Cetuximab alleviates neuropathic pain despite tumour progression. *BMJ Case Rep.* 2012:bcr1220115374. 10.1136/bcr.12.2011.5374 22707700 PMC3387481

[B95] Kersten C. Cameron M. G. Mjaland S. (2013). Epithelial growth factor receptor (EGFR)-inhibition for relief of neuropathic pain-A case series. *Scand. J. Pain* 4 3–7. 10.1016/j.sjpain.2012.11.011 29913887

[B96] Kersten C. Cameron M. G. Bailey A. G. Fallon M. T. Laird B. J. Paterson V. (2019). Relief of neuropathic pain through epidermal growth factor receptor inhibition: A randomized proof-of-concept trial. *Pain Med.* 20 2495–2505. 10.1093/pm/pnz101 31106835

[B97] Kersten C. Cameron M. G. Laird B. Mjaland S. (2015). Epidermal growth factor receptor-inhibition (Egfr-I) in the treatment of neuropathic pain. *Br. J. Anaesth.* 115 761–767. 10.1093/bja/aev326 26475804

[B98] Kilpatrick L. E. Hill S. J. (2021). Transactivation of G protein-coupled receptors (GPCRS) and receptor tyrosine kinases (RTKs): Recent insights using luminescence and fluorescence technologies. *Curr. Opin. Endocr. Metab. Res.* 16 102–112. 10.1016/j.coemr.2020.10.003 33748531 PMC7960640

[B99] Kim S. H. Chung J. M. (1992). An experimental model for peripheral neuropathy produced by segmental spinal nerve ligation in the rat. *Pain* 50 355–363. 10.1016/0304-3959(92)90041-9 1333581

[B100] Koo J. W. Lobo M. K. Chaudhury D. Labonte B. Friedman A. Heller E. (2014). Loss of BDNF signaling in D1R-expressing NAc neurons enhances morphine reward by reducing GABA inhibition. *Neuropsychopharmacology* 39 2646–2653. 10.1038/npp.2014.118 24853771 PMC4207344

[B101] Koo J. W. Mazei-Robison M. S. Chaudhury D. Juarez B. Laplant Q. Ferguson D. (2012). BDNF is a negative modulator of morphine action. *Science* 338 124–128. 10.1126/science.1222265 23042896 PMC3547365

[B102] Kutlar A. (2013). Glee-ful for sickle cell pain? *Blood* 122 1846–1847. 10.1182/blood-2013-07-510982 24030255

[B103] Lau E. K. Trester-Zedlitz M. Trinidad J. C. Kotowski S. J. Krutchinsky A. N. Burlingame A. L. (2011). Quantitative encoding of the effect of a partial agonist on individual opioid receptors by multisite phosphorylation and threshold detection. *Sci. Signal.* 4:ra52. 10.1126/scisignal.2001748 21868358 PMC3625704

[B104] Lemel L. Lane J. R. Canals M. (2020). GRKs as key modulators of opioid receptor function. *Cells* 9:2400. 10.3390/cells9112400 33147802 PMC7692057

[B105] Lemmon M. A. Schlessinger J. (2010). Cell signaling by receptor tyrosine kinases. *Cell* 141 1117–1134. 10.1016/j.cell.2010.06.011 20602996 PMC2914105

[B106] Li L. Chen S. R. Chen H. Wen L. Hittelman W. N. Xie J. D. (2016). Chloride homeostasis critically regulates synaptic NMDA receptor activity in neuropathic pain. *Cell Rep.* 15 1376–1383. 10.1016/j.celrep.2016.04.039 27160909 PMC4871741

[B107] Li Y. Eitan S. Wu J. Evans C. J. Kieffer B. Sun X. (2003). Morphine induces desensitization of insulin receptor signaling. *Mol. Cell Biol.* 23 6255–6266. 10.1128/MCB.23.17.6255-6266.2003 12917346 PMC180943

[B108] Li Z. Jia X. Peng X. Gao F. (2020). The Interaction between spinal PDGFRbeta and mu opioid receptor in the activation of microglia in morphine-tolerant rats. *J. Pain Res.* 13 1803–1810. 10.2147/JPR.S255221 32765055 PMC7381827

[B109] Liu S. Liu W. T. Liu Y. P. Dong H. L. Henkemeyer M. Xiong L. Z. (2011). Blocking EphB1 receptor forward signaling in spinal cord relieves bone cancer pain and rescues analgesic effect of morphine treatment in rodents. *Cancer Res.* 71 4392–4402. 10.1158/0008-5472.CAN-10-3870 21555368

[B110] Loh H. H. Liu H. C. Cavalli A. Yang W. Chen Y. F. Wei L. N. (1998). Mu opioid receptor knockout in mice: Effects on ligand-induced analgesia and morphine lethality. *Brain Res. Mol. Brain Res.* 54 321–326. 10.1016/S0169-328X(97)00353-7 9555078

[B111] Lopez-Bellido R. Puig S. Huang P. J. Tsai C. R. Turner H. N. Galko M. J. (2019). Growth factor signaling regulates mechanical nociception in flies and vertebrates. *J. Neurosci.* 39 6012–6030. 10.1523/JNEUROSCI.2950-18.2019 31138657 PMC6650988

[B112] Lynch J. J. III Wade C. L. Zhong C. M. Mikusa J. P. Honore P. (2004). Attenuation of mechanical allodynia by clinically utilized drugs in a rat chemotherapy-induced neuropathic pain model. *Pain* 110 56–63. 10.1016/j.pain.2004.03.010 15275752

[B113] Macey T. A. Bobeck E. N. Hegarty D. M. Aicher S. A. Ingram S. L. Morgan M. M. (2009). Extracellular signal-regulated kinase 1/2 activation counteracts morphine tolerance in the periaqueductal gray of the rat. *J. Pharmacol. Exp. Therap.* 331 412–418. 10.1124/jpet.109.152157 19684256 PMC2775267

[B114] Manglik A. Lin H. Aryal D. K. Mccorvy J. D. Dengler D. Corder G. (2016). Structure-based discovery of opioid analgesics with reduced side effects. *Nature* 537 185–190. 10.1038/nature19112 27533032 PMC5161585

[B115] Mansour A. Fox C. A. Burke S. Akil H. Watson S. J. (1995a). Immunohistochemical localization of the cloned mu opioid receptor in the rat CNS. *J. Chem. Neuroanat.* 8 283–305. 10.1016/0891-0618(95)00055-C 7669273

[B116] Mansour A. Watson S. J. Akil H. (1995b). Opioid receptors: Past, present and future. *Trends Neurosci.* 18 69–70. 10.1016/0166-2236(95)80024-V 7537414

[B117] Mansour A. Fox C. A. Burke S. Meng F. Thompson R. C. Akil H. (1994a). Mu, delta, and kappa opioid receptor mRNA expression in the rat CNS: An in situ hybridization study. *J. Comp. Neurol.* 350 412–438. 10.1002/cne.903500307 7884049

[B118] Mansour A. Fox C. A. Thompson R. C. Akil H. Watson S. J. (1994b). mu-Opioid receptor mRNA expression in the rat CNS: Comparison to mu-receptor binding. *Brain Res.* 643 245–265. 10.1016/0006-8993(94)90031-0 8032920

[B119] Mansour A. Khachaturian H. Lewis M. E. Akil H. Watson S. J. (1988). Anatomy of CNS opioid receptors. *Trends Neurosci.* 11 308–314. 10.1016/0166-2236(88)90093-8 2465635

[B120] Mao J. Price D. D. Mayer D. J. (1995). Mechanisms of hyperalgesia and morphine tolerance: A current view of their possible interactions. *Pain* 62 259–274. 10.1016/0304-3959(95)00073-2 8657426

[B121] Martin L. J. Smith S. B. Khoutorsky A. Magnussen C. A. Samoshkin A. Sorge R. E. (2017). Epiregulin and EGFR interactions are involved in pain processing. *J. Clin. Invest.* 127 3353–3366. 10.1172/JCI87406 28783046 PMC5669538

[B122] Martini L. Whistler J. L. (2007). The role of mu opioid receptor desensitization and endocytosis in morphine tolerance and dependence. *Curr.Opin. Neurobiol.* 17 556–564. 10.1016/j.conb.2007.10.004 18068348

[B123] Masuo Y. Morita M. Oka S. Ishido M. (2004). Motor hyperactivity caused by a deficit in dopaminergic neurons and the effects of endocrine disruptors: A study inspired by the physiological roles of PACAP in the brain. *Regul. Pept.* 123 225–234. 10.1016/j.regpep.2004.05.010 15518916

[B124] Matthes H. W. Maldonado R. Simonin F. Valverde O. Slowe S. Kitchen I. (1996). Loss of morphine-induced analgesia, reward effect and withdrawal symptoms in mice lacking the mu-opioid-receptor gene. *Nature* 383 819–823. 10.1038/383819a0 8893006

[B125] Mayer D. J. Mao J. Holt J. Price D. D. (1999). Cellular mechanisms of neuropathic pain, morphine tolerance, and their interactions. *Proc. Natl. Acad. Sci. U.S.A.* 96 7731–7736. 10.1073/pnas.96.14.7731 10393889 PMC33610

[B126] Mcconalogue K. Grady E. F. Minnis J. Balestra B. Tonini M. Brecha N. C. (1999). Activation and internalization of the mu-opioid receptor by the newly discovered endogenous agonists, endomorphin-1 and endomorphin-2. *Neuroscience* 90 1051–1059. 10.1016/S0306-4522(98)00514-4 10218804 PMC4472477

[B127] Mckay M. Morrison D. (2007). Integrating signals from RTKs to ERK/MAPK. *Oncogene* 26 3113–3121. 10.1038/sj.onc.1210394 17496910

[B128] Mclaughlin J. P. Chavkin C. (2001). Tyrosine phosphorylation of the mu-opioid receptor regulates agonist intrinsic efficacy. *Mol. Pharmacol.* 59 1360–1368. 10.1124/mol.59.6.1360 11353794

[B129] Mir H. R. Miller A. N. Obremskey W. T. Jahangir A. A. Hsu J. R. (2019). Confronting the opioid crisis: Practical pain management and strategies: AOA 2018 critical issues symposium. *J. Bone Joint Surg. Am.* 101:e126. 10.2106/JBJS.19.00285 31800430

[B130] Miyatake M. Rubinstein T. J. Mclennan G. P. Belcheva M. M. Coscia C. J. (2009). Inhibition of EGF-induced ERK/Map kinase-mediated astrocyte proliferation by mu opioids: Integration of G protein and beta-arrestin 2-dependent pathways. *J. Neurochem.* 110 662–674. 10.1111/j.1471-4159.2009.06156.x 19457093 PMC3236703

[B131] Molina-Munþoz T. Romero-Aìvila M. A. T. Garciìa-Saìinz J. A. (2006). Insulin-like growth factor-I induces α1B-adrenergic receptor phosphorylation through Gβγ and epidermal growth factor receptor transactivation. *Mol. Endocrinol.* 20 2773–2783. 10.1210/me.2006-0090 16803866

[B132] Moryl N. Obbens E. A. Ozigbo O. H. Kris M. G. (2006). Analgesic effect of gefitinib in the treatment of non-small cell lung cancer. *J. Support Oncol.* 4:111. 16553135

[B133] Mouledous L. Diaz M. F. Gutstein H. B. (2007). Extracellular signal-regulated kinase (ERK) inhibition does not prevent the development or expression of tolerance to and dependence on morphine in the mouse. *Pharmacol. Biochem. Behav.* 88 39–46. 10.1016/j.pbb.2007.07.002 17764731 PMC2151045

[B134] Murnion B. P. (2018). Neuropathic pain: Current definition and review of drug treatment. *Aust. Prescr.* 41:60. 10.18773/austprescr.2018.022 29921999 PMC6003018

[B135] Nair A. B. Jacob S. (2016). A simple practice guide for dose conversion between animals and human. *J. Basic Clin. Pharm.* 7:27. 10.4103/0976-0105.177703 27057123 PMC4804402

[B136] Narita M. Usui A. Narita M. Niikura K. Nozaki H. Khotib J. (2005). Protease-activated receptor-1 and platelet-derived growth factor in spinal cord neurons are implicated in neuropathic pain after nerve injury. *J. Neurosci.* 25 10000–10009. 10.1523/JNEUROSCI.2507-05.2005 16251448 PMC6725566

[B137] Navarro B. Kennedy M. E. Velimirović B. Bhat D. Peterson A. S. Clapham D. E. (1996). Nonselective and Gβγ-insensitive weaver K+ channels. *Science* 272 1950–1953. 10.1126/science.272.5270.1950 8658170

[B138] Noma T. Lemaire A. Prasad S. V. N. Barki-Harrington L. Tilley D. G. Chen J. (2007). β-arrestin–mediated β 1-adrenergic receptor transactivation of the Egfr confers cardioprotection. *J. Clin. Invest.* 117 2445–2458. 10.1172/JCI31901 17786238 PMC1952636

[B139] Oligny-Longpré G. Corbani M. Zhou J. Hogue M. Guillon G. Bouvier M. (2012). Engagement of β-arrestin by transactivated insulin-like growth factor receptor is needed for V2 vasopressin receptor-stimulated ERK1/2 activation. *Proc. Natl Acad. Sci. U.S.A.* 109 E1028–E1037. 10.1073/pnas.1112422109 22493236 PMC3340024

[B140] Overland A. C. Insel P. A. (2015). Heterotrimeric G proteins directly regulate Mmp14/membrane type-1 matrix metalloprotease: A novel mechanism for GPCR-EGFR transactivation. *J. Biol. Chem.* 290 9941–9947. 10.1074/jbc.C115.647073 25759388 PMC4400366

[B141] Pattinson K. T. (2008). Opioids and the control of respiration. *Br. J. Anaesth.* 100 747–758. 10.1093/bja/aen094 18456641

[B142] Paul A. K. Smith C. M. Rahmatullah M. Nissapatorn V. Wilairatana P. Spetea M. (2021). Opioid analgesia and opioid-induced adverse effects: A review. *Pharmaceuticals (Basel)* 14:1091. 10.3390/ph14111091 34832873 PMC8620360

[B143] Pawson T. (2004). Specificity in signal transduction: From phosphotyrosine-Sh2 domain interactions to complex cellular systems. *Cell* 116 191–203. 10.1016/S0092-8674(03)01077-8 14744431

[B144] Peregud D. I. Yakovlev A. A. Stepanichev M. Y. Onufriev M. V. Panchenko L. F. Gulyaeva N. V. (2016). Expression of BDNF and TRKB phosphorylation in the rat frontal cortex during morphine withdrawal are no dependent. *Cell Mol. Neurobiol.* 36 839–849. 10.1007/s10571-015-0267-6 26346883 PMC11482428

[B145] Petäjä-Repo U. E. Hogue M. Laperriere A. Bhalla S. Walker P. Bouvier M. (2001). Newly synthesized human δ opioid receptors retained in the endoplasmic reticulum are retrotranslocated to the cytosol, deglycosylated, ubiquitinated, and degraded by the proteasome. *J. Biol. Chem.* 276 4416–4423. 10.1074/jbc.M007151200 11054417

[B146] Phamduong E. Rathore M. K. Crews N. R. D’angelo A. S. Leinweber A. L. Kappera P. (2014). Acute and chronic mu opioids differentially regulate thrombospondins 1 and 2 isoforms in astrocytes. *ACS Chem. Neurosci.* 5 106–114. 10.1021/cn400172n 24304333 PMC3930990

[B147] Pickard A. S. Lee T. A. (2021). Combating the opioid epidemic in the United States. *Drugs Context* 10. 10.7573/dic.2021-10-7 34970324 PMC8687109

[B148] Pierce K. L. Luttrell L. M. Lefkowitz R. J. (2001). New mechanisms in heptahelical receptor signaling to mitogen activated protein kinase cascades. *Oncogene* 20 1532–1539. 10.1038/sj.onc.1204184 11313899

[B149] Posa L. Accarie A. Noble F. Marie N. (2016). Methadone reverses analgesic tolerance induced by morphine pretreatment. *Int. J. Neuropsychopharmacol.* 19:yv108. 10.1093/ijnp/pyv108 26390873 PMC4966270

[B150] Povsic T. J. Kohout T. A. Lefkowitz R. J. (2003). β-Arrestin1 mediates insulin-like growth factor 1 (Igf-1) activation of phosphatidylinositol 3-kinase (Pi3K) and anti-apoptosis. *J. Biol. Chem.* 278 51334–51339. 10.1074/jbc.M309968200 14534298

[B151] Przewlocki R. Przewlocka B. (2001). Opioids in chronic pain. *Eur. J. Pharmacol.* 429 79–91. 10.1016/S0014-2999(01)01308-5 11698029

[B152] Puig S. Gutstein H. B. (2017). Opioids: Keeping the good, eliminating the bad. *Nat. Med.* 23 272–273. 10.1038/nm.4277 28134927 PMC6602584

[B153] Puig S. Donica C. L. Gutstein H. B. (2020b). EGFR Signaling causes morphine tolerance and mechanical sensitization in rats. *eNeuro* 7. 10.1523/ENEURO.0460-18.2020 32111605 PMC7218007

[B154] Puig S. Barker K. E. Szott S. R. Kann P. T. Morris J. S. Gutstein H. B. (2020a). Spinal opioid tolerance depends upon platelet-derived growth factor receptor-beta signaling, not mu-opioid receptor internalization. *Mol. Pharmacol.* 98 487–496. 10.1124/mol.120.119552 32723769 PMC7562976

[B155] Quasthoff S. Hartung H. P. (2002). Chemotherapy-induced peripheral neuropathy. *J. Neurol.* 249 9–17. 10.1007/PL00007853 11954874

[B156] Raehal K. M. Bohn L. M. (2011). The role of beta-arrestin2 in the severity of antinociceptive tolerance and physical dependence induced by different opioid pain therapeutics. *Neuropharmacology* 60 58–65. 10.1016/j.neuropharm.2010.08.003 20713067 PMC2981657

[B157] Raehal K. M. Schmid C. L. Groer C. E. Bohn L. M. (2011). Functional selectivity at the mu-opioid receptor: Implications for understanding opioid analgesia and tolerance. *Pharmacol. Rev.* 63 1001–1019. 10.1124/pr.111.004598 21873412 PMC3186080

[B158] Ragunathrao V. A. B. Anwar M. Akhter M. Z. Chavez A. Natarajan V. Lakshmikanthan S. (2019). Sphingosine-1-phosphate receptor 1 activity promotes tumor growth by amplifying Vegf-Vegfr2 angiogenic signaling. *Cell Rep.* 29 3472–3487.e4. 10.1016/j.celrep.2019.11.036 31825830 PMC6927555

[B159] Rezamohammadi F. Rahmani M. Ghanbari A. Khaleghian A. Miladi-Gorji H. (2020). Bdnf receptor antagonism during the induction of morphine dependence exacerbates the severity of physical dependence and ameliorates psychological dependence in rats. *Neurosci. Lett.* 737:135332. 10.1016/j.neulet.2020.135332 32860885

[B160] Rivat C. Sar C. Mechaly I. Leyris J.-P. Diouloufet L. Sonrier C. (2018). Inhibition of neuronal Flt3 receptor tyrosine kinase alleviates peripheral neuropathic pain in mice. *Nat. Commun.* 9 1–13. 10.1038/s41467-018-03496-2 29531216 PMC5847526

[B161] Robinson D. R. Wu Y.-M. Lin S.-F. (2000). The protein tyrosine kinase family of the human genome. *Oncogene* 19 5548–5557. 10.1038/sj.onc.1203957 11114734

[B162] Roskoski R. Jr. (2018). The role of small molecule platelet-derived growth factor receptor (PDGFR) inhibitors in the treatment of neoplastic disorders. *Pharmacol. Res.* 129 65–83. 10.1016/j.phrs.2018.01.021 29408302

[B163] Russo S. J. Bolanos C. A. Theobald D. E. Decarolis N. A. Renthal W. Kumar A. (2007). Irs2-Akt pathway in midbrain dopamine neurons regulates behavioral and cellular responses to opiates. *Nat. Neurosci.* 10 93–99. 10.1038/nn1812 17143271

[B164] Saito Y. Haendeler J. Hojo Y. Yamamoto K. Berk B. C. (2001). Receptor heterodimerization: Essential mechanism for platelet-derived growth factor-induced epidermal growth factor receptor transactivation. *Mol. Cell Biol.* 21 6387–6394. 10.1128/MCB.21.19.6387-6394.2001 11533228 PMC99786

[B165] Saloner B. Mcginty E. E. Beletsky L. Bluthenthal R. Beyrer C. Botticelli M. (2018). A public health strategy for the opioid crisis. *Public Health Rep.* 133 24S–34S. 10.1177/0033354918793627 30426871 PMC6243441

[B166] Sasahara M. Fries J. W. Raines E. W. Gown A. M. Westrum L. E. Frosch M. P. (1991). PDGF B-chain in neurons of the central nervous system, posterior pituitary, and in a transgenic model. *Cell* 64 217–227. 10.1016/0092-8674(91)90223-L 1986868

[B167] Savage D. G. Antman K. H. (2002). Imatinib mesylate–a new oral targeted therapy. *N. Engl. J. Med.* 346 683–693. 10.1056/NEJMra013339 11870247

[B168] Scherrer G. Imamachi N. Cao Y. Q. Contet C. Mennicken F. O’donnell D. (2009). Dissociation of the opioid receptor mechanisms that control mechanical and heat pain. *Cell* 137 1148–1159. 10.1016/j.cell.2009.04.019 19524516 PMC3683597

[B169] Schlessinger J. (2000). New roles for Src kinases in control of cell survival and angiogenesis. *Cell* 100 293–296. 10.1016/S0092-8674(00)80664-9 10676810

[B170] Schmid C. L. Kennedy N. M. Ross N. C. Lovell K. M. Yue Z. Morgenweck J. (2017). Bias factor and therapeutic window correlate to predict safer opioid analgesics. *Cell* 171 1165–1175.e13. 10.1016/j.cell.2017.10.035 29149605 PMC5731250

[B171] Shi X. Miller J. S. Harper L. J. Poole R. L. Gould T. J. Unterwald E. M. (2014). Reactivation of cocaine reward memory engages the AKT/Gsk3/MTOR signaling pathway and can be disrupted by Gsk3 inhibition. *Psychopharmacology* 231 3109–3118. 10.1007/s00213-014-3491-8 24595501 PMC4110417

[B172] Sim E. H. Yang I. A. Wood-Baker R. Bowman R. V. Fong K. M. (2018). Gefitinib for advanced non-small cell lung cancer. *Cochrane Database Syst. Rev.* 1:Cd006847. 10.1002/14651858.CD006847.pub2 29336009 PMC6491254

[B173] Singla N. K. Skobieranda F. Soergel D. G. Salamea M. Burt D. A. Demitrack M. A. (2019). Apollo-2: A randomized, placebo and active-controlled phase iii study investigating oliceridine (Trv 130), a G protein–biased ligand at the μ−opioid receptor, for management of moderate to severe acute pain following abdominoplasty. *Pain Pract.* 19 715–731. 10.1111/papr.12801 31162798 PMC6851842

[B174] Soergel D. G. Subach R. A. Burnham N. Lark M. W. James I. E. Sadler B. M. (2014). Biased agonism of the μ-opioid receptor by Trv130 increases analgesia and reduces on-target adverse effects versus morphine: A randomized, double-blind, placebo-controlled, crossover study in healthy volunteers. *Pain* 155 1829–1835. 10.1016/j.pain.2014.06.011 24954166

[B175] Spliet W. G. Aronica E. Ramkema M. Witmer A. N. Schlingemann R. O. De Jong J. M. (2004). Immunohistochemical localization of vascular endothelial growth factor receptors–1, –2 and –3 in human spinal cord: Altered expression in amyotrophic lateral sclerosis. *Neuropathol. Appl. Neurobiol.* 30 351–359. 10.1111/j.1365-2990.2003.00543.x 15305980

[B176] Stankovic Stojanovic K. Thioliere B. Garandeau E. Lecomte I. Bachmeyer C. Lionnet F. (2011). Chronic myeloid leukaemia and sickle cell disease: Could imatinib prevent vaso-occlusive crisis? *Br. J. Haematol.* 155 271–272. 10.1111/j.1365-2141.2011.08670.x 21488859

[B177] Sternini C. Spann M. Anton B. Keith D. E. Jr. Bunnett N. W. Von Zastrow M. (1996). Agonist-selective endocytosis of mu opioid receptor by neurons in vivo. *Proc Natl Acad Sci U.S.A.* 93 9241–9246. 10.1073/pnas.93.17.9241 8799185 PMC38626

[B178] Stuart G. L. Shorey R. C. France C. R. Macfie J. Bell K. Fortner K. B. (2018). Empirical studies addressing the opioid epidemic: An urgent call for research. *Subst. Abuse* 12:1178221818784294. 10.1177/1178221818784294 30127614 PMC6090487

[B179] Sun N. Zhang X. Guo S. Le H. T. Zhang X. Kim K.-M. (2018). Molecular mechanisms involved in epidermal growth factor receptor-mediated inhibition of dopamine D3 receptor signaling. *Biochim. Biophys. Acta* 1865 1187–1200. 10.1016/j.bbamcr.2018.06.001 29885323

[B180] The Council of Economic Advisers (2017). *The underestimated cost of the opioid crisis.* Washington, DC: The Council of Economic Advisers.

[B181] Trafton J. A. Abbadie C. Marek K. Basbaum A. I. (2000). Postsynaptic signaling via the [mu]-opioid receptor: Responses of dorsal horn neurons to exogenous opioids and noxious stimulation. *J. Neurosci.* 20 8578–8584. 10.1523/JNEUROSCI.20-23-08578.2000 11102461 PMC6773096

[B182] Tsai R.-Y. Tai Y.-H. Tzeng J.-I. Cherng C.-H. Yeh C.-C. Wong C.-S. (2009). Ultra-low dose naloxone restores the antinociceptive effect of morphine in pertussis toxin-treated rats by reversing the coupling of μ-opioid receptors from Gs-protein to coupling to Gi-protein. *Neuroscience* 164 435–443. 10.1016/j.neuroscience.2009.08.015 19682558

[B183] Ugolini G. Marinelli S. Covaceuszach S. Cattaneo A. Pavone F. (2007). The function neutralizing anti-TrkA antibody Mnac13 reduces inflammatory and neuropathic pain. *Proc. Natl Acad. Sci. U.S.A.* 104 2985–2990. 10.1073/pnas.0611253104 17301229 PMC1815293

[B184] Viscusi E. R. Skobieranda F. Soergel D. G. Cook E. Burt D. A. Singla N. (2019). Apollo-1: A randomized placebo and active-controlled phase Iii study investigating oliceridine (Trv130), a G protein-biased ligand at the μ-opioid receptor, for management of moderate-to-severe acute pain following bunionectomy. *J. Pain Res.* 12:927. 10.2147/JPR.S171013 30881102 PMC6417853

[B185] Wang Y. Barker K. Shi S. Diaz M. Mo B. Gutstein H. B. (2012). Blockade of PDGFR-beta activation eliminates morphine analgesic tolerance. *Nat. Med.* 18 385–387. 10.1038/nm.2633 22344297 PMC3296828

[B186] Wang Z. Ma W. Chabot J.-G. Quirion R. (2009). Cell-type specific activation of p38 and Erk mediates calcitonin gene-related peptide involvement in tolerance to morphine-induced analgesia. *FASEB J.* 23 2576–2586. 10.1096/fj.08-128348 19299480

[B187] Weber M. L. Chen C. Li Y. Farooqui M. Nguyen J. Poonawala T. (2013). Morphine stimulates platelet-derived growth factor receptor-beta signalling in mesangial cells in vitro and transgenic sickle mouse kidney in vivo. *Br. J. Anaesth.* 111 1004–1012. 10.1093/bja/aet221 23820675 PMC3828056

[B188] Werner M. H. Nanney L. B. Stoscheck C. M. King L. E. (1988). Localization of immunoreactive epidermal growth factor receptors in human nervous system. *J. Histochem. Cytochem.* 36 81–86. 10.1177/36.1.3275713 3275713

[B189] Wetzker R. Bohmer F. D. (2003). Transactivation joins multiple tracks to the ERK/MAPK cascade. *Nat. Rev. Mol. Cell Biol.* 4 651–657. 10.1038/nrm1173 12923527

[B190] Whistler J. L. (2012). Examining the role of mu opioid receptor endocytosis in the beneficial and side-effects of prolonged opioid use: From a symposium on new concepts in mu-opioid pharmacology. *Drug Alcohol Depend.* 121 189–204. 10.1016/j.drugalcdep.2011.10.031 22226706 PMC4224378

[B191] Whistler J. L. Von Zastrow M. (1998). Morphine-activated opioid receptors elude desensitization by β-arrestin. *Proc. Natl. Acad. Sci. U.S.A.* 95 9914–9919. 10.1073/pnas.95.17.9914 9707575 PMC21436

[B192] Whistler J. L. Chuang H. H. Chu P. Jan L. Y. Von Zastrow M. (1999). Functional dissociation of mu opioid receptor signaling and endocytosis: Implications for the biology of opiate tolerance and addiction. *Neuron* 23 737–746. 10.1016/S0896-6273(01)80032-5 10482240

[B193] Williams J. T. Ingram S. L. Henderson G. Chavkin C. Von Zastrow M. Schulz S. (2013). Regulation of mu-opioid receptors: Desensitization, phosphorylation, internalization, and tolerance. *Pharmacol. Rev.* 65 223–254. 10.1124/pr.112.005942 23321159 PMC3565916

[B194] Xia W. S. Peng Y. N. Tang L. H. Jiang L. S. Yu L. N. Zhou X. L. (2014). Spinal ephrinB/EphB signalling contributed to remifentanil-induced hyperalgesia via NMDA receptor. *Eur. J. Pain* 18 1231–1239. 10.1002/j.1532-2149.2014.00478.x 24737575 PMC4232047

[B195] Xu Y. Lv X.-F. Cui C.-L. Ge F.-F. Li Y.-J. Zhang H.-L. (2012). Essential role of Nr2B-containing NMDA receptor–ERK pathway in nucleus accumbens shell in morphine-associated contextual memory. *Brain Res. Bull.* 89 22–30. 10.1016/j.brainresbull.2012.06.012 22776695

[B196] Yang Y. Sun Y. Hu R. Yan J. Wang Z. Li W (2021). Morphine promotes microglial activation by upregulating the EGFR/ERK signaling pathway. *PLoS One* 9:e0256870. 10.1371/journal.pone.0256870 34520454 PMC8439491

[B197] Zamora-Martinez E. R. Edwards S. (2014). Neuronal extracellular signal-regulated kinase (ERK) activity as marker and mediator of alcohol and opioid dependence. *Front. Integr. Neurosci.* 8:24. 10.3389/fnint.2014.00024 24653683 PMC3949304

[B198] Zamponi G. W. Bourinet E. Nelson D. Nargeot J. Snutch T. P. (1997). Crosstalk between G proteins and protein kinase C mediated by the calcium channel α1 subunit. *Nature* 385 442–446. 10.1038/385442a0 9009192

[B199] Zhao H. Wu G. Cao X. (2013). Egfr dependent subcellular communication was responsible for morphine mediated Ac superactivation. *Cell Signal.* 25 417–428. 10.1016/j.cellsig.2012.10.016 23142605

[B200] Zhou J. Ma R. Jin Y. Fang J. Du J. Shao X. (2021). Molecular mechanisms of opioid tolerance: From opioid receptors to inflammatory mediators. *Exp. Therap. Med.* 22 1–8. 10.3892/etm.2021.10437 34345286 PMC8311239

